# Variant predictions in congenital adrenal hyperplasia caused by mutations in CYP21A2

**DOI:** 10.3389/fphar.2022.931089

**Published:** 2022-10-05

**Authors:** Mayara J. Prado, Rodrigo Ligabue-Braun, Arnaldo Zaha, Maria Lucia Rosa Rossetti, Amit V. Pandey

**Affiliations:** ^1^ Graduate Program in Cell and Molecular Biology, Universidade Federal do Rio Grande do Sul (UFRGS), Porto Alegre, Brazil; ^2^ Center for Biotechnology, Universidade Federal do Rio Grande do Sul (UFRGS), Porto Alegre, Brazil; ^3^ Translational Hormone Research, Department of Biomedical Research, University of Bern, Bern, Switzerland; ^4^ Pediatric Endocrinology Unit, Department of Pediatrics, University Children’s Hospital Bern, Bern, Switzerland; ^5^ Departament of Pharmacosciences, Universidade Federal de Ciências da Saúde de Porto Alegre (UFCSPA), Porto Alegre, Brazil; ^6^ Graduate Program in Molecular Biology Applied to Health, Universiade Luterana do Brasil (ULBRA), Canoas, Brazil

**Keywords:** online prediction, CYP21A2, mutation analysis, CAH, steroid metabolism, pathogenicity prediction tools

## Abstract

CYP21A2 deficiency represents 95% of congenital adrenal hyperplasia (CAH) cases, a group of genetic disorders that affect steroid biosynthesis. The genetic and functional analysis provide critical tools to elucidate complex CAH cases. One of the most accessible tools to infer the pathogenicity of new variants is *in silico* prediction. Here, we analyzed the performance of *in silico* prediction tools to categorize missense single nucleotide variants (SNVs) of *CYP21A2*. SNVs of *CYP21A2* characterized *in vitro* by functional assays were selected to assess the performance of online single and meta predictors. SNVs were tested separately or in combination with the related phenotype (severe or mild CAH form). In total, 103 SNVs of *CYP21A2* (90 pathogenic and 13 neutral) were used to test the performance of 13 single-predictors and four meta-predictors. All SNVs associated with the severe phenotypes were well categorized by all tools, with an accuracy of between 0.69 (PredictSNP2) and 0.97 (CADD), and Matthews’ correlation coefficient (MCC) between 0.49 (PoredicSNP2) and 0.90 (CADD). However, SNVs related to the mild phenotype had more variation, with the accuracy between 0.47 (S3Ds&GO and MAPP) and 0.88 (CADD), and MCC between 0.18 (MAPP) and 0.71 (CADD). From our analysis, we identified four predictors of *CYP21A2* variant pathogenicity with good performance, CADD, ConSurf, DANN, and PolyPhen2. These results can be used for future analysis to infer the impact of uncharacterized SNVs in *CYP21A2*.

## 1 Introduction

One of the most common autosomal recessive genetic disorders is the impairment of the steroid 21-hydroxylase (CYP21A2). The CYP21A2 deficiency represents about 95% of cases in congenital adrenal hyperplasia (CAH), a group of enzymatic disorders that affect cortisol biosynthesis. The CYP21A2 enzyme is a member of the cytochrome P450 superfamily (CYPs) and catalyzes the conversion of 17α-hydroxyprogesterone (17OHP) into 11-deoxycortisol and progesterone into 11-deoxycorticosterone. Other enzymes subsequently convert these steroids into cortisol and aldosterone (Miller and Auchus, 2011).

Clinically, the CYP21A2 deficiency in humans has a wide spectrum of phenotypes, from severe to mild or asymptomatic (New et al., 2013; Witchel, 2017). The classic severe CAH has salt-wasting (SW) and simple-virilizing (SV) forms. The classical SW form has no enzyme activity and is related to severe virilization and electrolyte imbalance. In contrast, the classical SV form has enough residual enzyme activity to prevent adrenal crisis (Witchel, 2017). Mild CAH is the non-classical (NC) form of CAH and has CYP21A2 activity associated with hyperandrogenism and mild late-onset CAH (New et al., 2013). Furthermore, there is a relatively good genotype-phenotype correlation for CYP21A2 deficiency, which allows the categorization of variants according to the residual enzyme activity (obtained from *in vitro* studies) and their expected phenotype (New et al., 2013). The classical CAH (CL) has less than 10% of wild-type (WT) enzyme activity in 95% of the cases, while the NC form has an activity of between 10 and 78% of the WT in 90% of the cases, as reported by Simonetti et al., (Simonetti et al., 2018).

The *CYP21A2* gene is a tandemly arranged module (RCCX: *RP-C4-CYP21*-*TNX*) and shows 96–98% of sequence identity with its pseudogene, *CYP21A1P* (Rodrigues et al., 1987). These features make the *CYP21A2* gene analysis a complex endeavor, with many different types of mutations—from single nucleotide variants (SNVs) to genetic rearrangements—and further complicated by the fact that most carriers have compound heterozygous mutations (New et al., 2013). However, only ten mutations described in the general population are sampled by CYP21A2 deficiency screening programs. Whole gene sequence analysis by Sanger sequencing is an alternative method in exceptional cases due to the cost and time-consuming nature of such studies (Stenson et al., 2017; Baumgartner-Parzer et al., 2020).

So far, with the whole *CYP21A2* gene sequenced, genetic studies have reported more than 1,300 variants in the *CYP21A2* gene. Out of the 230 variants reported as affecting human health, 153 are missense variants (Simonetti et al., 2018). The advancement of next-generation sequencing (NGS) to analyze a large number of genes has facilitated the detection of rare single nucleotide variants (SNVs). A few years ago, this technology was not applied to screen the *CYP21A2* gene defects due to its high sequence identity with *CYP21A2P,* which hampers the proper analysis of this genomic region (Rodrigues et al., 1987). However, recently, some groups have found alternative ways to perform NGS for the *CYP21A2* gene through a combination with other methods, such as multiplex ligation-dependent probe amplification (Gangodkar et al., 2021; Lee et al., 2021). These genetic analysis strategies of the *CYP21A2* gene with NGS technology represent a promising tool for the future, opening the window to identify new variants while improving the diagnosis of CYP21A2 deficiency and establishing a more reliable estimate of mutation frequencies.

The gold standard for the characterization of new CYP21A2 variants is the *in vitro* functional assay. However, this approach takes too much time, and it is not a viable option for the analysis of all the new variants detected by sequencing studies. One of the most accessible tools to predict the pathogenicity of variants is *in-silico* analysis, which usually has free access, a friendly interface, and provides quick results. Many online predictors are available that have different features and approaches, from single characteristic analysis to meta-predictors with different compositions and algorithms. Some studies have shown the general performance of these tools against a whole database with few predictors (Hicks et al., 2011; Tang et al., 2020). However, studies with variants on protein-specific analysis showed that general analysis results cannot be extrapolated for all proteins as each protein has unique characteristics, which is a key limitation of predictor programs (Pshennikova et al., 2019; Hart et al., 2020; Montenegro et al., 2021). Therefore, it is essential to be careful when choosing the prediction tools and to consider their variable accuracies for each gene (Tang et al., 2020).

**FIGURE 1:**
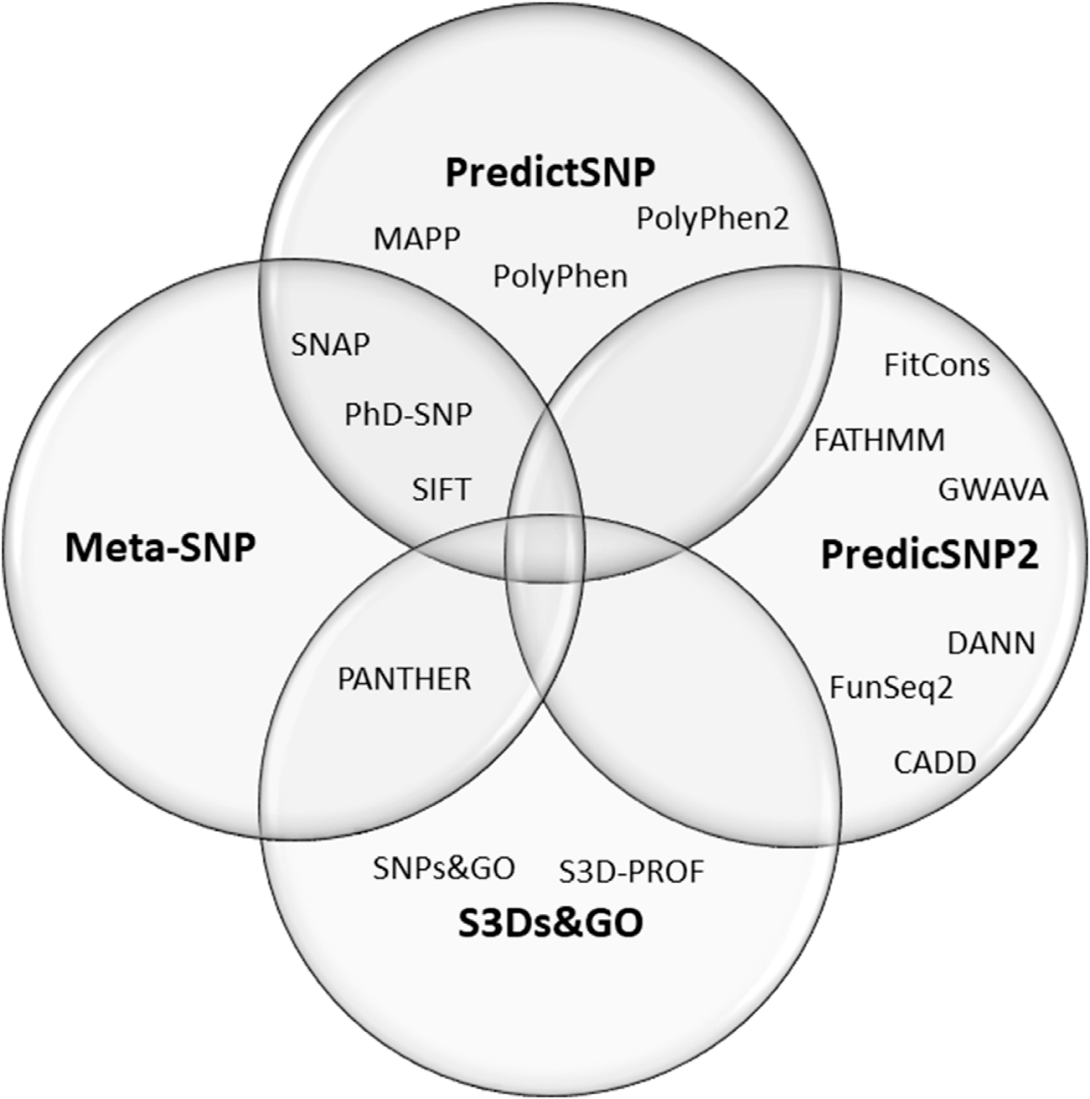
Composition of the four meta-predictors studied. The PredictSNP algorithm comprises the outputs of six single-predictors, the PredictSNP2 of six, the Meta-SNP of 4, and the S3Ds&GO of three predictors.

Here we have done a meta-analysis of the performance of online predictor tools to classify missense SNVs of CYP21A2. Missense SNV is the most common group of variants in the human genome, one at every kilobase. In the *CYP21A2* gene, this type of SNV represents about 60% of the CYP21A2 variants in The Human Gene Mutation Database (HGMD, RRID:SCR_001888) (Stenson et al., 2017) and 65% of those affecting human health (Simonetti et al., 2018). Additionally, missense SNV is one of the hardest variant types to interpret (Khan and Vihinen, 2007; Simonetti et al., 2018; Pignatelli et al., 2019). In total, we analyzed 17 predictors with multiple algorithms, approaches, and datasets. Thirteen of these were based on single features: CADD (RRID:SCR_018393) (van der Velde et al., 2017), ConSurf (RRID:SCR_002320) (Ashkenazy et al., 2016), DANN (Quang et al., 2015), FATHMM (Shihab et al., 2013), MAPP (RRID:SCR_014375) (Stone and Sidow, 2005), MutPred2 (RRID:SCR_010778) (Pejaver et al., 2020), PANTHER-PSEP (RRID: SCR_005145) (Tang and ThomasPANTHER-PSEP, 2016), PhD-SNP^g^ (RRID: SCR_010782) (Capriotti and FariselliPhD-SNPg, 2017), PolyPhen-2 (RRID:SCR_013189) (Adzhubei et al., 2013), PROVEN (RRID: SCR_002182) (Choi et al., 2012), SIFT (RRID:SCR_012813) (Vaser et al., 2016), SNAP2 (RRID:SCR_002127) (Hecht et al., 2015), and SNPs&GO (RRID:SCR_005788) (Calabrese et al., 2009). Four meta-predictors: PredictSNP (Bendl et al., 2014), PredictSNP2 (Bendl et al., 2016), Meta-SNP (Capriotti et al., 2013), and S3Ds&GO (Capriotti and Altman, 2011)) (Figure 1). We excluded nonsense and frameshift variants from our analysis since they have specific settings in some predictors which are not applied for single amino acid substitution and a high agreement ratio between tools.

## 2 Results

### 2.1 Data of the selected SNVs

From variants in the *CYP21A2* gene reported in the literature and databases, we selected missense SNVs with clinical significance, using the criteria described in Section 4.1. We obtained 96 valid SNVs out of 299 missense variants in the list (Simonetti et al., 2018), 85 out of 614 missense variants in dbSNP, 66 out of 459 missense variants in Ensembl, 45 out of 71 missense variants in GeneCards, 47 out of 83 missense variants in ClinVar, 26 in OMIM, and 81 found in the UniProt database.

By removing SNVs that were duplicated and with no functional characterization, we obtained 103 SNVs, 51 classified as classical, 39 as non-classical, and 13 as neutral. The SNVs selected with the respective enzyme activity are described in Supplementary Table S1. All studies presented the CYP21A2 activity measured by the hydroxylation of 17OHP, while 86 also measured progesterone hydroxylation. Mutations of the CL group have a mean enzyme activity for the 17OHP hydroxylation of 1.5 ± 2 (SD)% and the progesterone hydroxylation of 1.3 ± 1.6%. While mutations of the NC group have 17OHP hydroxylation activity of 42.9 ± 23% and progesterone hydroxylation activity of 37.4 ± 21%. Finally, mutations in the neutral group have 17OHP hydroxylation activity of 100.17 ± 11% and progesterone hydroxylation activity of 94.1 ± 8.25%.

### 2.2 General analysis of mutation groups

**FIGURE 2:**
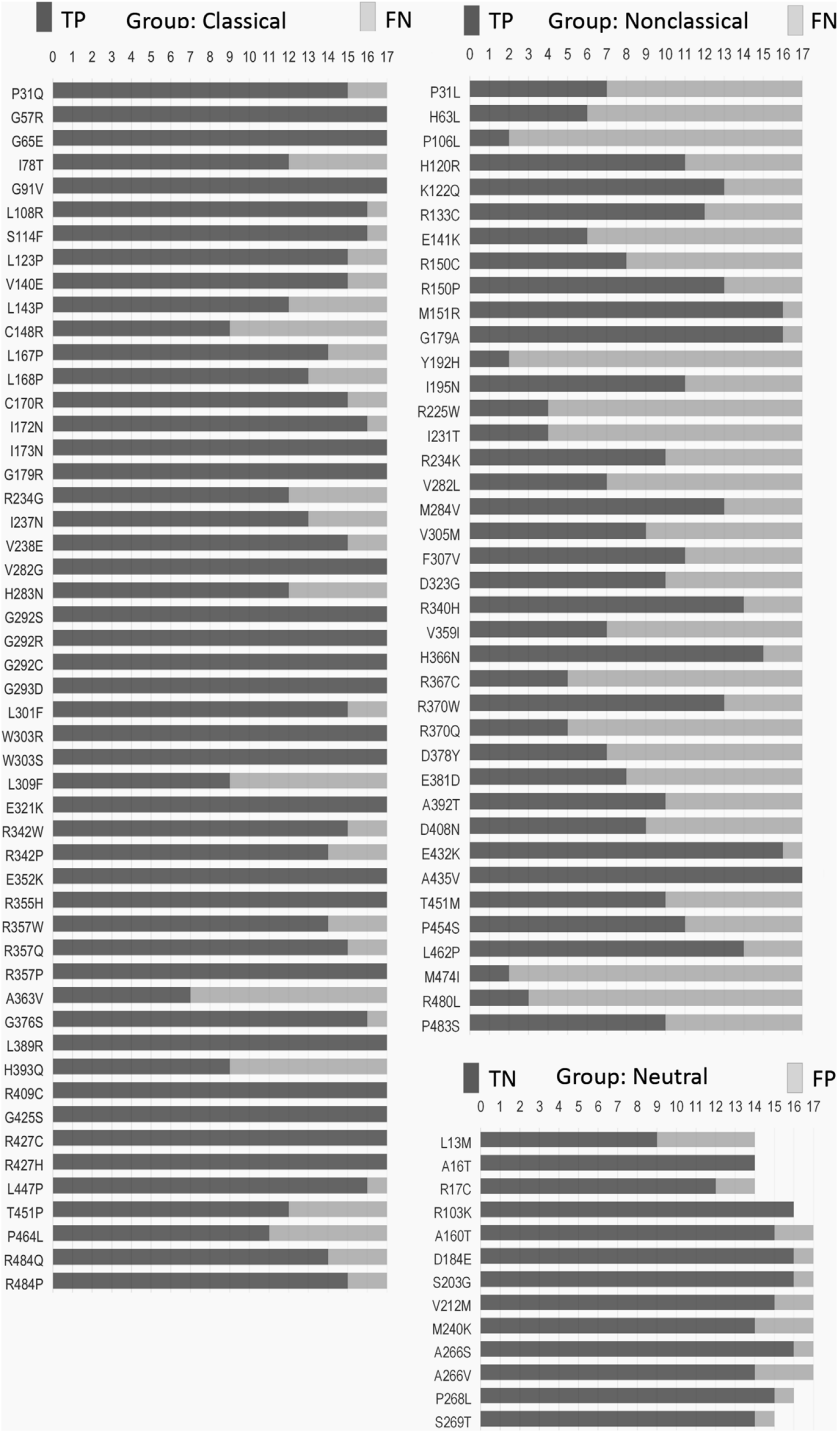
Frequency of hit and miss obtained for each SNV by mutation group. Each SNV (vertical list) was analyzed by seventeen predictors (horizontal measurement) performed with the default setting for missense mutation. Please, refer to Supplementary Table S2–S4 for details. TP, true positive; TN, true negative; FN, false negative; FP, false positive.

We obtained 22 SNVs in the *CYP21A2* gene with the correct prediction for all tested predictors, although the exact number was incorrectly predicted for at least half of them (Figure 2). There was no SNV, as wrongly predicted by all predictors. We compared the hit and miss by the 17 predictors for all SNVs affecting CYP21A2 activity, the CAH group, and all the non-pathogenic SNVs of the neutral group. We showed that 24% (22 of 90) SNVs from the CAH group obtained the correct score by all predictors, while 23% (21 of 90) were wrongly predicted by at least nine tools. The neutral group got two of its 13 SNVs (15.4%) rightly predicted by all and one by nine predictors. Moreover, we divided the SNV of the CAH group into the CL and NC groups. We got 37% (out of 51 SNVs) from the CL and 2.6% (of 39 SNVs) from the NC group of SNVs correctly predicted by all tools. While 5.9% and 46% of CL and NC groups, respectively, were wrongly predicted by nine tools.

Some critical amino acid positions have two or three pathogenic replacements (e.g., p.P31Q/L, p.V282G/L, and p.R357W/Q/P) (Figure 2). Two tools of prediction were able to identify the critical amino acid position for all of those double/triple mutants: CADD and ConSurf (Supplementary Table S2–S4). As those variants have the functional data available, CADD was able to use that information together with gene annotation, epigenetic and evolutionary data, outputting the right prediction. On the other hand, ConSurf obtained the same result using structural, phylogenetic, and evolutionary data.

### 2.3 Performance of predictors to identify SNVs detrimental to CYP21A2 activity

We analyzed the performance of 17 predictors to identify the 90 SNVs that affect CYP21A2 function against the 13 SNVs with a neutral effect (Table 1). All the predictors tested obtained a good PPV rate (>0.90). However, only CADD (0.73) and DANN (0.56) showed negative predictive values (NPVs) higher than 0.5. The PANTHER-PSEP found no result for the NPV, as it could not identify benign variants.

**TABLE 1:** Performance of 17 programs to predict the effect SNVs in the *CYP21A2*. We performed the analysis with 103 functionally characterized variants, 90 damaging the protein functionality, and 13 neutral—Color scores from blue (good result) to yellow (not good).

Predictors	TP	FN	TN	FP	PPV	NPV	Se	Sp	Ac	MCC
Meta-SNP^a^	60	30	13	0	1.00	0.30	0.67	1.00	0.71	0.45
PredictSNP^a^	58	32	13	0	1.00	0.29	0.64	1.00	0.69	0.43
PredictSNP2^a^	44	46	13	0	1.00	0.22	0.49	1.00	0.55	0.33
S3Ds&GO^a^	58	32	8	0	1.00	0.20	0.64	1.00	0.67	0.36
CADD	86	4	11	2	0.98	0.73	0.96	0.85	0.94	0.75
ConSurf	79	11	9	1	0.99	0.45	0.88	0.90	0.88	0.58
DANN	83	7	9	4	0.95	0.56	0.92	0.69	0.89	0.56
FATHMM	62	28	13	0	1.00	0.32	0.69	1.00	0.73	0.47
MAPP	54	36	10	2	0.96	0.22	0.60	0.83	0.63	0.28
MutPred2	62	28	13	0	1.00	0.32	0.69	1.00	0.73	0.47
PANTHER-PSEP	90	0	0	9	0.91	nr	1.00	0.00	0.91	Nr
PhD-SNPg	62	28	12	1	0.98	0.30	0.69	0.92	0.72	0.42
PolyPhen2-HumVar	79	11	12	1	0.99	0.52	0.88	0.92	0.88	0.64
PROVEAN	66	24	13	0	1.00	0.35	0.73	1.00	0.77	0.51
SIFT	70	20	11	2	0.97	0.35	0.78	0.85	0.79	0.45
SNP2	59	31	13	0	1.00	0.30	0.66	1.00	0.70	0.44
SNPs&GO	54	36	13	0	1.00	0.27	0.60	1.00	0.65	0.40

a meta-predictor. Nr, no result; PPV, positive predictive value; NPV, negative predictive value; Se, sensitivity; Sp, specificity; Ac, accuracy; MCC, Matthews’ correlation coefficient test.

**FIGURE 3:**
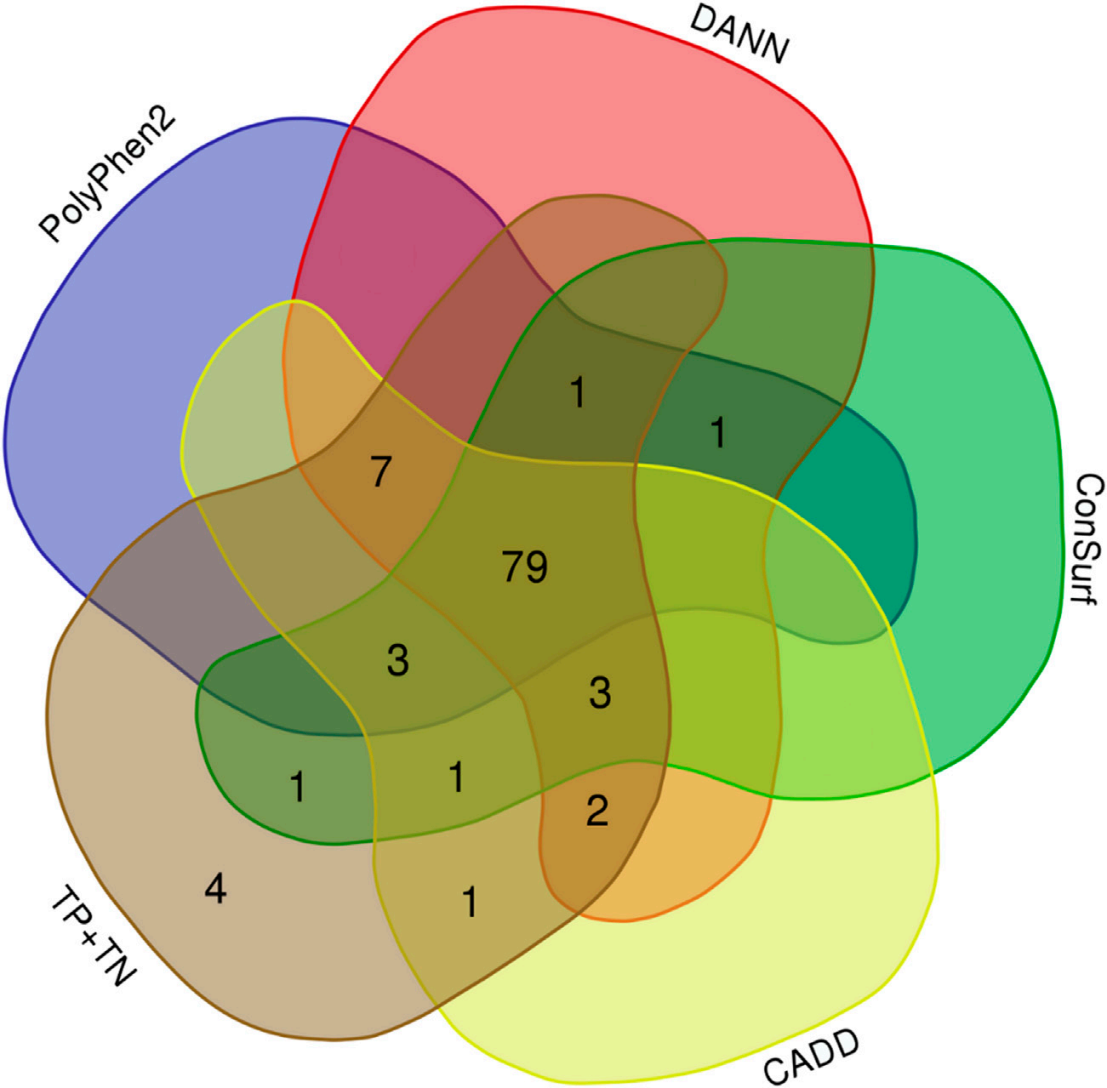
Overlaps of the hit for four predictors with good-excellent performance for SNVs on CYP21A2. Total, indicates all 103 SNVs tested. (Image generated with https://bioinformatics.psb.ugent.be/webtools/Venn/).

We obtained both sensitivity and specificity higher than 0.8 for three predictors, CADD (sensitivity, e, = 0.96 and specificity, sp, = 0.85), ConSurf (se = 0.88 and sp = 0.90), and PolyPhen-2 (se = 0.87 and sp = 0.85). Moreover, five predictors obtained accuracy between excellent and good: CADD (0.94), PANTHER-PSEP (0.91), DANN (0.89), ConSurf (0.88), and PolyPhen-2 (0.86) (Table 1). The Matthews’ correlation coefficient (MCC) test showed positive values for that of almost all predictors (except by PANTHER-PSEP), being five of them with an MCC >0.5 (Table 1). The greatest performance, a value closer to +1, was obtained by CADD (0.75), followed by ConSurf (0.58), PolyPhen-2 (0.57), DANN (0.56), and PROVEN (0.51). Figure 3 shows a Venn diagram of the four predictors with better accuracy and MCC values.

### 2.4 Performance of predictors to identify SNVs affecting the specific CAH groups

We analyzed the performance of the selected predictors to identify 51 SNVs of the CL group and 39 SNVs of the NC group against 13 SNVs with a neutral effect (Table 2). Seventeen predictors obtained an excellent positive predictive value (PPV) rate (>0.90) for the SNV CL group and 15 for the SNV NC group. Four tools obtained excellent-good (>0.8) negative predictive values (NPV) values for the CL group: CADD (1.0), DANN (0.9), PolyPhen-2 (0.85), and ConSurf (0.82). However, for the NC group, only CADD (0.73), DANN (0.6), and PolyPhen-2 (0.52) showed NPV >0.5. Taking the sensitivity and specificity balance, we obtained 12 tools with excellent-good values (>0.8) for the CL group. However, for the NC group, only CADD (se = 0.9 and sp = 0.85) obtained both sensitivity and specificity with excellent-good values. The accuracy was excellent for seven predictors in the CL group: CADD (0.97), ConSurf (0.95), PolyPhen-2 and PROVEN (0.94), DANN and MutPred2 (0.92), and Meta-SNP (0.91); and good for four tools in the NC group: CADD (0.88), DANN and PANTHER-PSEP (0.81), and ConSurf (0.8).

**TABLE 2:** Performance of 17 programs for the specific CYP21A2 groups. We predict the effect of SNVs in the CYP21A2 by dividing them by the two levels of protein damage: severe (classical mutation, CL group) and mild (non-classical mutation, NC group). We performed the analysis with 103 SNVs of known effect, 51 being CL, 39 NC, and 13 neutral. Color score from blue (good result) to yellow (not good).

Specifics groups	PPV		NPV		Se		Sp		Ac		MCC	
	CL	NC	CL	NC	CL	NC	CL	NC	CL	NC	CL	NC
Meta-SNP^a^	1.00	1.00	0.68	0.35	0.88	0.38	1.00	1.00	0.91	0.54	0.78	0.37
PredictSNP^a^	1.00	1.00	0.65	0.34	0.86	0.36	1.00	1.00	0.89	0.52	0.75	0.35
PredictSNP2^a^	1.00	1.00	0.39	0.33	0.61	0.33	1.00	1.00	0.69	0.50	0.49	0.33
S3Ds&GO^a^	1.00	1.00	0.53	0.24	0.86	0.36	1.00	1.00	0.88	0.47	0.68	0.29
CADD	0.96	0.95	1.00	0.73	1.00	0.90	0.85	0.85	0.97	0.88	0.90	0.71
ConSurf	0.98	0.97	0.82	0.50	0.96	0.77	0.90	0.90	0.95	0.80	0.83	0.56
DANN	0.93	0.89	0.90	0.60	0.98	0.85	0.69	0.69	0.92	0.81	0.75	0.51
FATHMM	1.00	1.00	0.52	0.45	0.76	0.59	1.00	1.00	0.81	0.69	0.63	0.51
MAPP	0.95	0.88	0.48	0.29	0.78	0.36	0.83	0.83	0.79	0.47	0.51	0.18
MutPred2	1.00	1.00	0.72	0.36	0.90	0.41	1.00	1.00	0.92	0.56	0.81	0.38
PANTHER-PSEP	0.85	0.81	nr	nr	1.00	1.00	0.00	0.00	0.85	0.81	nr	nr
PhD-SNPg	0.98	0.95	0.57	0.39	0.82	0.51	0.92	0.92	0.84	0.62	0.64	0.38
PolyPhen2-HumVar	0.96	0.94	0.85	0.52	0.96	0.74	0.85	0.85	0.94	0.77	0.81	0.52
PROVEAN	1.00	1.00	0.76	0.39	0.92	0.49	1.00	1.00	0.94	0.62	0.84	0.44
SIFT	0.96	0.92	0.69	0.42	0.90	0.62	0.85	0.85	0.89	0.67	0.70	0.40
SNP2	1.00	1.00	0.59	0.37	0.82	0.44	1.00	1.00	0.86	0.58	0.70	0.40
SNPs&GO	1.00	1.00	0.59	0.33	0.82	0.31	1.00	1.00	0.86	0.48	0.70	0.32

a meta-predictor. Nr, no result; PPV, positive predictive value; NPV, negative predictive value; Se, sensitivity; Sp, specificity; Ac, accuracy; MCC, Matthews’ correlation coefficient test.

Finally, the MCC test with positive values was obtained by almost all predictors (except for PANTHER-PSEP). The MCC was higher than 0.5 for 16 predictors in the CL group and five in the NC group (Table 2). For the CL and NC groups, the same predictor, CADD, got the MCC value closer to +1, with MCC = 0.9 for the CL group and MCC = 0.71 for the NC group.

## 3 Discussion

Genetics analysis is an essential approach for elucidating complex *CYP21A2* deficiency cases, mainly to confirm asymptomatic carriers and unfollow false-positive cases (Pignatelli et al., 2019; Baumgartner-Parzer et al., 2020). Therefore, fast and accessible tools to infer variants’ pathogenicity are essential to quickly deduce the harm of unknown variants. *In silico* prediction is one of the most accessible tools to infer the pathogenicity of SNVs. Here, for the first time, we analyzed the performance of *in silico* prediction tools to discriminate between pathogenic and neutral variants of *CYP21A2*. We focus on the performance of 13 single predictors and four meta predictors chosen accordingly to the popularity and performance of free-access programs. Although some programs are based on the same data set, each one has particular features (e.g., evolutionary, epigenetic, functional, gene annotation, protein structure), algorithms (e.g., machine learning, matrix of effect, alignment), and databanks (e.g., ClinVar, SwissVar, UniProt, UniRef90, HumVar). Furthermore, they also differ in the input data (protein change, nucleotide change, chromosomal position, and accession number of the gene). The 17 programs chosen in the present study are composed of distinct combinations of features, databanks, and algorithms (Supplementary Table S5). All of them were able to identify pathogenic variants. Nonetheless, only PANTHER-PSEP could not distinguish neutral variants, which is unacceptable for testing variants of the *CYP21A2*. Moreover, all tools showed better performance with variants of the CL group than the NC group, as expected, since the CL group gathers the most harmful variants.

Our databank for performance tests comprises all missense variants of CYP21A2 that are functionally characterized. With this strategy, we could get a more realistic result on the prediction evaluation. However, the number of variants was imbalanced between the two categories, with 90 pathogenic and 13 neutral. Therefore, the primary statistics data considered for the performance evaluation were the accuracy and MCC, which consider all values—true positive (TP), true negative (TN), false positive (FP), and false negative (FN) (Vihinen, 2012; Chicco et al., 2021). As sensitivity and specificity are calculated with half of the information, they cannot represent all the performance by themselves, so we considered the sensitivity-specificity balance. Additionally, we calculated PPV and NPV but, as both are more sensitive to data disbalance, they were not considered for the program performance (Vihinen, 2012).

The main feature assessed by most single predictors tested is the evolutionary data since residue conservation over time can indicate critical residues for the protein function. Four of the tools tested use only this feature for the prediction calculation, FATHMM (Shihab et al., 2013), PhD-SNPg (Capriotti and FariselliPhD-SNPg, 2017), PROVEAN (Choi et al., 2012), and SIFT (Vaser et al., 2016). A similar performance was obtained between these four tools, with a fair accuracy ranging from 0.73 (FATHMM) to 0.79 (SIFT), and MCC from 0.42 (PhD-SNPg) to 0.51 (PROVEAN). Additionally, SIFT had the most excellent sensitivity-specificity balance between programs, similar to the performance shown previously (Vaser et al., 2016). In another study, Montenegro *et al.* (Simonetti et al., 2018), with *HSD17B3*, *NR5A1*, *AR*, and *LHCGR* genes, SIFT and PROVEAN also had the same performance, with an accuracy of 0.74–0.75, and MCC of 0.5. In yet another study (Pshennikova et al., 2019), SIFT and PROVEAN showed the best results among nine programs tested for *GJB2*, *GJB6*, and *GJB3* genes, with an accuracy of 0.89, while FATHMM produced a large number of erroneous predictions with an accuracy of 0.33. FATHMM also had poor performance in another study (Montenegro et al., 2021), with an accuracy of 0.56 and a MCC of 0.04.

Changes in the secondary and tertiary structures by missense mutations are likely to affect protein activity (Ashkenazy et al., 2016). Therefore, it is no surprise that the second most evaluated feature is structural information, being present in ConSurf (Ashkenazy et al., 2016), MutPred2 (Pejaver et al., 2020), PolyPhen2 (Hicks et al., 2011), and SNAP2 (Hecht et al., 2015). In addition, ConSurf includes phylogenetics relationships, MutPred2 functional proprieties, and SNAP2 uses a matrix of effect probabilities with a neural network method (Hecht et al., 2015; Ashkenazy et al., 2016; Pejaver et al., 2020). While PolyPhen2 has two trained datasets as options, HumVar and HumDir. The first trained dataset is suggested for diagnostics of Mendelian diseases, which requires variants with a drastic difference effect (Adzhubei et al., 2013). Moreover, PolyPhen2 has a low dependency on the sequence alignment employed (Hicks et al., 2011). PolyPhen2 showed good prediction performance (ac = 0.88, MCC = 0.64), with the same sensitivity but a better sensitivity-specificity balance than reported in (Hicks et al., 2011). ConSurf obtained a similar performance with the setting used in the test (ac = 0.88, MCC = 0.58). However, we cannot compare the ConSurf performance with other studies since there is no default setting for the alignment sequence, database, and algorithms available, as mentioned by ConSurf’s developers (Ashkenazy et al., 2016). MutPred2 and SNAP2, both had sensitivity-specificity imbalances of 1.4 and 1.5-folds, respectively, and almost the same fair performance. However, these values were relatively better than reported for MutPred2 by (Pejaver et al., 2020) with the ClinVar and UniProt databases and SNAP2 by (Hecht et al., 2015) with a databank with more than 9,500 variants from human genes.

For meta predictors, which work with many databases and combine outputs from other predictors to generate their own, we expected to obtain one of the best performances. However, counterintuitively, our study showed an intermediate performance compared with the single predictors tested, and the number of tools combined was not related to the prediction improvement. Meta-SNP and PredictSNP performance were better than PredictSNP2 and S3Ds&GO. Furthermore, compared with the developer tests, we obtained for Meta-SNP (Capriotti et al., 2013) and PredictSNP (Bendl et al., 2014) similar accuracy, while the performance for PredictSNP2 (Bendl et al., 2016) and S3Ds&GO (Capriotti and Altman, 2011) was lower. Meta-SNP and PredictSNP share three single predictors, PhD-SNP, SNAP, and SIFT.

Therefore, for the *CYP21A2* variants tested, CADD presented the best performance to categorize the pathogenicity of the missense variants, with an overall accuracy of 0.94 (CL 0.97; NC 0.88) and MCC of 0.75 (CL 0.9, NC 0.71). The specificity (0.85) and sensitivity (0.9) also reached a good balance. Interestingly, the accuracy and specificity obtained for CYP21A2 were even higher than reported by the software developers (van der Velde et al., 2017) with the ClinVar database, which were 0.85 and 0.57, respectively. ConSurf, DANN, and PolyPhen-2 showed similar performance, giving the second-best results according to the accuracy (CAH group 0.86–0.89; CL 0.92–0.95; NC 0.77–0.81) and MCC (CAH group 0.56–0.58; CL 0.75–0.83; NC 0.51–0.56) values. The sensitivity and specificity for ConSurf and PolyPhen2 were well balanced, while DANN had 1.3-fold less specificity than sensitivity. The original article of DANN (Quang et al., 2015) presents only the area under the curve (AUC) ROC, which was 0.95 using the ClinVar database for the performance test. PolyPhen2 showed a better sensitivity-specificity balance for the *CYP21A2* variants than the values presented by (Hicks et al., 2011), testing the tool with gene-specific mutations (*BRCA1*, *MSH2*, *MLH1*, and *TP52*). We obtained a sensitivity similar to a previous analysis (Hicks et al., 2011), but the specificity was lower, at 0.85 and 0.60, respectively.

Considering the individual errors of the four predictors with the greatest performance for the CYP21A2 variants analyzed in this study, CADD had six errors, ConSurf 12, DANN 11, and PolyPhen2 14. However, computing their predictions together, we would have four false results from 103 missense SNVs in the *CYP21A2*, one neutral (p.L13M), and three pathogenic from the NC phenotype group (p.P106L, p.R225W, and p.M474I). The variant p.P106L was correctly categorized by SNAP2 and PANTHER-PSEP. In turn, PROVEAN (Choi et al., 2012) could type the other three variants correctly, even with a lower sensitivity value than the other four tools, mainly for the pathogenic SNVs of the NC group (0.49). Nonetheless, we obtained an intermediated performance with PROVEAN (ac = 0.77; MCC = 0.51), which could be because it uses the neighborhood sequences as input, which can be a trick for enzymes since they have some residues with high conservation, making critical connections between variable residues. In comparison using the developer’s test (Choi et al., 2012) with the UniProt database (se = 0.78; sp = 0.79), we had imbalanced sensitivity-specificity performance, with similar overall sensitivity (0.73) and higher specificity (1.0).

One of the limitations associated with this analysis is the unbalanced number of variants in each group analyzed due to the limited number of variants characterized. In order to diminish this issue, only statistical approaches considering the whole data were used for the performance comparison, as recommended in the literature (Vihinen, 2012; Chicco et al., 2021). Another limitation was the selection of the tools available online since each predictor has a specific input such as chromosomal version, the number of variants per input, and data input type. For that reason, we established parameters to choose a limited number of predictors based on the features and literature citations.

## 4 Materials and methods

### 4.1 SNVs selection and categorization

To select *CYP21A2* missense SNVs with clinical significance, we used the list of variants reported to affect human health, as reviewed by Simonetti *et al.* (Simonetti et al., 2018) Complementarily, we searched for SNVs reported by dbSNP, Ensembl, and GeneCards, applying the following filters when present: “missense,” “clinical significance,” “pathogenetic,” or “benign,” “without conflicting interpretation”, and “human or homo sapiens”. We excluded nonsense and frameshift mutations. In addition, we performed a cross-check of the databases with original articles or reviews to remove variants without enzyme activity data available.

To standardize the effect of the SNVs selected, we categorized them into three groups according to the CYP21A2 activity observed by Simonetti *et al.* (Simonetti et al., 2018) for at least one of the steroid substrates: i) CL group has SNVs with the CYP21A2 activity level <10% relative to WT; ii) NC group has SNVs with the activity level between >10% and <78% relative to WT; and iii) the neutral group has SNV with the enzyme activity >78% relative to WT. The CAH group is composed of all SNVs from the CL and NC. The mean and standard deviation (SD) of enzyme activity were calculated for each steroid and mutation group.

### 4.2 Selection of predictor tools

To choose predictors with different features, we reviewed the literature for software applied to *in silico* analysis of SNPs or SNVs. Predictors used in more than two studies by different research groups or significant performance in a large study were selected. In addition, we filtered for tools with free access and online availability, thus not requiring local powerful computational resources. The characteristics of each predictor chosen are shown in Supplementary Table S5–S6 **.**

### 4.3 Data treatment

The default setting for missense mutation was used on all predictors. However, when there was no set instruction for that purpose highlighted for the program, we followed the developers’ recommendation from the tutorial or original paper. In addition, three scores were extracted indirectly from meta-predictors: MAPP (v.28.6.2005) and SIFT (v.4.0.4) scores were obtained from PredictSNP, and DANN (v.1.2) score from PredictSNP2. For statistical purposes, we standardized two variables for the outputs of all the predictors: “damage” for SNVs with the potential to affect CYP21A2 and “neutral” for SNVs with no or very low potential to affect the enzyme. The following outputs were standardized as “damage”: score >0.5 to Meta-SNP, SNP&GO, S3D&GO, MutPred2, FATHMM-MKL (weighted) and PhD-SNP^g^; “deleterious” message to PredictSNP, PredictSNP2, MAPP, and SIFT; score >0.9 to DANN; score > -2.5 to PROVEN; score >10 to CADD (GRCh38-v1.5–6); score >0.45 PolyPhen-2 (HumVar); score <0 to ConSurf; score >0 to SNAP2; and score >450 millions of years to PANTHER. Otherwise, we classified the outputs as “neutral".

### 4.4 Analytical parameters

We analyzed the performance of each predictor in two ways. First, to assess the performance to discriminate the effects of *CYP21A2* SNVs, we compared SNVs of the CAH group with the neutral group. Second, to get the number of hits and misses per group, we analyzed CL and NC groups separated from the neural group. We used Microsoft Excel for the data organization and, together with IBM SPSS Statistics software v.2.1, we performed the statistical analysis.

### 4.5 Statistical methods

We considered the TP result for correct “damage” prediction, TN for correct “neutral” prediction, FP for incorrect “neutral” prediction, and FN for incorrect “damage” prediction. We calculated the PPV to access the ratio of TP results for all positive results (Eq. 1), and the NPV to the ratio of TN for all negative results (Eq. 2).

$$PPV=\frac{TP}{TP+FP}$$ (1)

$$NPV=\frac{TN}{TN+FN}$$ (2)

The proportion of correct SNVs identified as harmful was assessed with the sensitivity (Se) equation (Eq. 3), while the correct neutral identification was assessed with the specificity (Sp) (Eq. 4). Besides that, we obtained the accuracy (Ac) by the ratio of true results (TP and TN) (Eq. 5). The accuracy was classified as excellent (0.9 < Ac < 1.0), good (0.8 < Ac < 0.9), fair (0.7 < Ac < 0.8), and not good (0.6 < Ac < 0.7).

$$Se=\frac{TP}{TP+FN}$$ (3)

$$Sp=\frac{TN}{TN+FP}$$ (4)

$$Ac=\frac{TP+TN}{TP+FP+TN+FN}$$ (5)

Finally, we applied the MCC to measure the two-class quality (harmful and neutral). This method is suitable for imbalanced data and has been used to evaluate *in silico* prediction approaches. MCC score ranges from 1 (perfect prediction) to -1 (total disagreement between the results predicted and observed), with 0 being no better than random prediction (Eq. 6) (Chicco et al., 2021).

$$MCC=\frac{TP\;X\;TN-FP\;X\;FN}{\sqrt{(TP+FP)(TP+FN)(TN+FP)\left(TN+FN\right)}}$$ (6)

## 5 Conclusion

The cost-effective and easy method of SNV analysis for CYP21A2 has important value as a screening tool, especially with a large number of genetic variants being available in massive genome projects. In the present study, we compared the abilities of 17 online and free tools for predicting the pathogenicity of SNVs on the *CYP21A2* gene, a highly complex gene. Based on a curated databank composed of SNVs on the CYP21A2 enzyme functionally characterized, we reported the four highest-performing predictor programs for characterizing the pathogenicity of the CL group—CADD, ConSurf, DANN, and PolyPhen2. One of them, CADD, also showed the best performance for identifying mild mutations from the NC group, followed by ConSurf and DANN. Therefore, according to those results, CADD, ConSurf, DANN, and PolyPhen2 are high-recommended to be run for the first screening of each uncharacterized CYP21A2 SNV. Moreover, there is a great probability of a missense variant of the CYP21A2 being “pathogenic” when at least two of those four tools obtain that result. These results may be applicable in the future analysis of new missense variants of *CYP21A2* and emphasize the relevance of using multiple predictors together.

## Data Availability

The original contributions presented in the study are included in the article/Supplementary Material; further inquiries can be directed to the corresponding authors.
