# Supplementary material for: Variant predictions in congenital adrenal hyperplasia caused by mutations in CYP21A2
Source: Front Pharmacol. 2022 Oct 5;13:931089. doi: 10.3389/fphar.2022.931089 (PMC9579345; doi:10.3389/fphar.2022.931089)
Supplement: Supplementary file 1 [file DataSheet1.docx]

***Supplementary Material***

**1 Supplementary Tables**

**Table S1**. List of the 103 single nucleotide variants (SNVs) on CYP21A2 gene selected to test the performance of predictor tools. SNVs are grouped into classical (enzyme activity < 10%), non-classical (between 10 and 78 %) and neutral (> 78 %) groups. The enzyme activity levels of both 21-hydroxylase substrates - 17-hydroxyprogesterone and progesterone - were obtained from the original paper of the functional characterization. The phenotype was obtained from either the same paper or the original description of the new SNV. ^a^ Shows the percentage of enzyme activity measured for the conversion of both 21-hydroxylase substrates, considering as 100 % the 21-hydroxylase wild type activity. 17OHP: 17-hydroxyprogesterone. SW: salt wasting. SV: simple-virilizing. NC: non-classical. ND: non-determinate.

|  |  | | | | Activity *in vitro* | | | | | | | |  | | | |  | | |
| --- | --- | --- | --- | --- | --- | --- | --- | --- | --- | --- | --- | --- | --- | --- | --- | --- | --- | --- | --- |
| Group | NP_000491.4 | | | | 17OHP^a^ | | SD (±) | | | Progesterone | | SD (±) | | | Phenotype | Publication | |  |  |
| CL | p.P31Q | | | | 0.2 | | 0.2 | | | 0 | | 0 | | | SW | [1] | |  |  |
|  | p.G57R | | | | 0.7 | | ND | | | 1.4 | | ND | | | SV | [2] | |  |  |
|  | p.G65E | | | | 0 | | ND | | | 0 | | ND | | | SW | [3] | |  |  |
|  | p.I78T | | | | 3 | | 2 | | | 5 | | 3 | | | SV | [4] | |  |  |
|  | p.G91V | | | | 0 | | ND | | | 0 | | ND | | | SW | [5] | |  |  |
|  | p.L108R | | | | 0.4 | | ND | | | 0.3 | | ND | | | SW | [2] | |  |  |
|  | p.S114F | | | | 4 | | 1 | | | 4 | | 2 | | | SV | [6] | |  |  |
|  | p.L123P | | | | 1.42 | | 2.13 | | | -1.86 | | 5.19 | | | SW | [7] | |  |  |
|  | p.V140E | | | | 0.7 | | 1.3 | | | 0.5 | | 0.6 | | | SW | [8] | |  |  |
|  | p.L143P | | | | 0.4 | |  | | | 0.4 | |  | | | SW | [2] | |  |  |
|  | p.C148R | | | | 4.3 | | 0.9 | | | 3.6ny | | 1.8 | | | SV-NC | [8] | |  |  |
|  | p.L167P | | | | 0.3 | | 0.06 | | | 0.4 | | 0.6 | | | SW | [9] | |  |  |
|  | p.L168P | | | | 0.7 | | ND | | | 0.4 | | ND | | | SW | [10] | |  |  |
|  | p.C170R | | | | 0.1 | | 0.02 | | | 0 | | 2 | | | SW | [11] | |  |  |
|  | p.I172N | | | | 0.7 | | 0.3 | | | 0.6 | | 0.03 | | | SV | [12] | |  |  |
|  | p.I173N | | | | 4.3 | | 1.7 | | | 4.4 | | 1.8 | | | SV | [10] | |  |  |
|  | p.G179R | | | | 0.4 | | 0.5 | | | 0 | | 0.6 | | | SW | [11] | |  |  |
|  | p.R234G | | | | 8 | | 2 | | | 2 | | 1 | | | SV-NC | [13] | |  |  |
|  | p.I237N | | | | 1 | | 0.7 | | | 2.4 | | 1.4 | | | SV | [14] | |  |  |
|  | p.V238E | | | | 0 | | 0 | | | 0.1 | | 0.3 | | | SW | [14] | |  |  |
|  | p.V282G | | | | 3.9 | | 1.7 | | | 3.9 | | 2 | | | SV | [15] | |  |  |
|  | p.H283N | | | | 1.6 | | 6 | | | 2.7 | | 5 | | | SW | [16] | |  |  |
|  | p.G292C | | | | 0 | | ND | | | 0 | | ND | | | SW | [5] | |  |  |
|  | p.G292R | | | | 0.5 | | 0.7 | | | 0.7 | | 0.2 | | | SW | [8] | |  |  |
|  | p.G292S | | | | 0.8 | | 0.4 | | | 0.8 | | 0.4 | | | SW | [17] | |  |  |
|  | p.G293D | | | | 0.5 | | 0.2 | | | 0.7 | | 0.4 | | | SW | [10] | |  |  |
|  | p.L301F | | | | 9.5 | | 6.4 | | | 4.4 | | 2.5 | | | SV | [15] | |  |  |
|  | p.W303S | | | | 3 | | 0.3 | | | 3 | | 0.5 | | | SV-NC | [18] | |  |  |
|  | p.W303R | | | | 0.1 | | 0.2 | | | 0 | | 0.5 | | | SW | [11] | |  |  |
|  | p.L309F | | | | 0.2 | | 0.3 | | | 0.1 | | 0.3 | | | SW | [8] | |  |  |
|  | p.E321K | | | | 4.6 | | 1.8 | | | 4.5 | | 2.6 | | | SV | [10] | |  |  |
|  | p.R342P | | | | 0.7 | | 0.3 | | | 0.7 | | 0.2 | | | SV | [12] | |  |  |
|  | p.R342W | | | | 5 | | 0.4 | | | 4 | | 3 | | | SV-NC | [13] | |  |  |
|  | p.E352K | | | | 1.1 | | 0.5 | | | 1.2 | | 0.3 | | | SV | [19] | |  |  |
|  | p.R355H | | | | 0 | | ND | | | 0 | | ND | | | SW | [5] | |  |  |
| .  Continuation (Table S1) |  | | | |  | |  | | |  | |  | | |  |  | |  |  |
|  | p.R357P | | | | 0.15 | | 0.3 | | | 0.15 | | 0.3 | | | SW | [20] | |  |  |
|  | p.R357Q | | | | 0.65 | | 0.44 | | | 1.1 | | 0.94 | | | SV | [20] | |  |  |
|  | p.R357W | | | | 0 | | ND | | | 0 | | ND | | | SW | [21] | |  |  |
|  | p.A363V | | | | 0 | | ND | | | 0 | | ND | | | SW | [3] | |  |  |
|  | p.G376S | | | | 1.6 | | 0.8 | | | 0.7 | | 0.7 | | | SW | [22] | |  |  |
|  | p.L389R | | | | 1.1 | | 0.6 | | | ND | | ND | | | SW | [23] | |  |  |
|  | p.H393Q | | 2.5 | | | | | 0.6 | | 2.2 | | 0.6 | | | SW | [24] | | |  |
|  | p.R409C | | 1.3 | | | | | 0.5 | |  | |  | | | SW | [2] | | |  |
|  | p.G425S | | 1.6 | | | | | 0.4 | | 2 | | 0.6 | | | SV | [10] | | |  |
|  | p.R427C | | 0 | | | | | 0.5 | | 0 | | 0.6 | | | SW | [11] | | |  |
|  | p.R427H | | 0.5 | | | | | 0.6 | | 0.4 | | 0.2 | | | SW-SV | [12] | | |  |
|  | p.L447P | | 0.5 | | | | | 0.6 | | 0 | | 0.1 | | | SW-SV | [12] | | |  |
|  | p.T451P | | 0.9 | | | | | ND | | 0.9 | | ND | | | SW | [6] | | |  |
|  | p.P464L | | 2.6 | | | | | 0.8 | | 3 | | 0.5 | | | SV | [25] | | |  |
|  | p.R484P | | 1 | | | | | 0.07 | | 2.2 | | 0.9 | | | SV | [17] | | |  |
|  | p.R484Q | | 1.1 | | | | | 0.7 | | 3.8 | | 1.9 | | | SV | [9] | | |  |
|  | Mean | | 1.52 | | | | |  | | 1.32 | |  | | |  |  | | |  |
|  | SD | | 2.00 | | | | |  | | 1.61 | |  | | |  |  | | |  |
| NC | p.P31L | | 13 | | | | | 0.2 | | 2 | | 0.6 | | | NC | [13] | | |  |
|  | p.H63L | | 44.5 | | | | | ND | | 20.7 | | ND | | | NC | [2] | | |  |
|  | p.P106L | | 62 | | | | | 9 | | 64 | | 12 | | | NC | [26] | | |  |
|  | p.H120R | | 31.6 | | | | | 8 | | 32.5 | | 7 | | | NC | [27] | | |  |
|  | p.K122Q | | 14 | | | | | 5 | | 19.5 | | 4 | | | NC | [28] | | |  |
|  | p.R133C | | 35.4 | | | | | 7.4 | | 15.5 | | 2.7 | | | NC | [29] | | |  |
|  | p.E141K | | 11.3 | | | | | 2.4 | | ND | | ND | | | SW | [23] | | |  |
|  | p.R150C | | 35.8 | | | | | 14.6 | | 47.3 | | 12.9 | | | NC | [29] | | |  |
|  | p.R150P | | 23.4 | | | | | 1.7 | | 16.9 | | 2 | | | NC | [30] | | |  |
|  | p.M151R | | 17.66 | | | | | 1.87 | | 4.57 | | 1.96 | | | NC | [7] | | |  |
|  | p.G179A | | 19 | | | | | ND | | ND | | ND | | | NC | [5] | | |  |
|  | p.Y192H | | 37.1 | | | | | 7 | | 25.8 | | 9 | | | NC | [16] | | |  |
|  | p.I195N | | 33.2 | | | | | 9 | | 46.7 | | 10 | | | NC | [27] | | |  |
|  | p.R225W | | 51.9 | | | | | 9 | | 45.6 | | 8 | | | NC | [31] | | |  |
|  | p.I231T | | 63.1 | | | | | 22.3 | | 70.6 | | 17 | | | NC | [10] | | |  |
|  | p.R234K | | 15 | | | | | ND | | 8.1 | | ND | | | SV-NC | [10] | | |  |
|  | p.V282L | | 18 | | | | | 3 | | 18 | | 5 | | | NC | [13] | | |  |
|  | p.M284V | | 16.2 | | | | | 9.3 | | 19 | | 6.8 | | | NC | [29] | | |  |
|  | p.V305M | | 46 | | | | | 18 | | 26 | | 10 | | | NC | [32] | | |  |
|  | p.F307V | | 63.23 | | | | | 5.5 | | 64.17 | | 7.98 | | | SV-NC | [33] | | |  |
|  | p.D323G | | 18 | | | | | 1.2 | | 27 | | 4.7 | | | NC | [18] | | |  |
|  | p.R340H | | 67.1 | | | | | 2.4 | | 45.8 | | 3.7 | | | NC | [34] | | |  |
|  | p.V359I | | 72 | | | | | 7 | | 34 | | 3 | | | NC | [35] | | |  |
|  | p.H366N | | 46.13 | | | | | 4.8 | | 57.77 | | 3.69 | | | NC | [33] | | |  |
|  | p.R367C | | 37 | | | | | 7 | | 28 | | 4 | | | NC | [13] | | |  |
|  | p.R370Q | | 82 | | | | | 6 | | 63 | | 4 | | | NC | [35] | | |  |
|  | p.R370W | | 45.8 | | | | | 1.8 | | 48.5 | | 17.1 | | | NC | [10] | | |  |
|  | p.D378Y | | 81 | | | | | 6 | | 58 | | 4 | | | NC | [35] | | |  |
|  | p.E381D | | 30 | | | | | ND | | ND | | ND | | | SW | [36] | | |  |
|  | p.A392T | | 38.7 | | | | | 9.5 | | 22.9 | | 4.7 | | | NC | [37] | | |  |
|  | p.D408N | | 72.7 | | | | | 7 | | 73.6 | | 10 | | | NC | [31] | | |  |
|  | p.E432K | | 26.2 | | | | | 3.8 | | 24.2 | | 7.4 | | | NC | [29] | | |  |
|  | p.A435V | | 14 | | | | | 2 | | 12 | | 6 | | | SV | [4] | | |  |
| . |  | |  | | | | |  | |  | |  | | |  |  | | |  |
|  | p.T451M | | 78 | | | | | 6 | | 43 | | 5 | | | NC | [6] | | |  |
|  | p.P454S | | 38 | | | | | ND | | 22.4 | | 3 | | | NC | [13] | | |  |
|  | p.L462P | | 55 | | | | | 8 | | 40 | | 2 | | | NC | [35] | | |  |
|  | p.M474I | | 85 | | | | | 7 | | 66 | | 12 | | | NC | [13] | | |  |
|  | p.R480L | | 75.5 | | | | | 15.7 | | 79.6 | | 12 | | | NC-Normal | [37] | | |  |
|  | p.P483S | | 61 | | | | | 6 | | 54 | | 2 | | | NC | [13] | | |  |
|  | Mean | | 42.94 | | | | |  | | 37.41 | |  | | |  |  | | |  |
|  | SD | | 22.59 | | | | |  | | 20.98 | |  | | |  |  | | |  |
| Neutral | | p.L13M | | 99 | | 1 | | | 100 | | 1 | | | Normal | | [6] | |  |  |
|  |  | p.A16T | | 100 | | 0 | | | 96 | | 6 | | | Normal- very mildNC | | [6] | |  |  |
|  |  | p.R17C | | 95 | | 3 | | | 81 | | 3 | | | Normal- very mildNC | | [6] | |  |  |
|  |  | p.R103K | | 119.7 | | 22.5 | | | ND | | ND | | | Normal | | [23] | |  |  |
|  |  | p.A160T | | 126.6 | | 29.9 | | | ND | | ND | | | Normal | | [23] | |  |  |
|  |  | p.D184E | | 100 | | ND | | | 100 | | ND | | | Normal | | [38] | |  |  |
|  |  | p.S203G | | 85 | | 2 | | | 81 | | 3 | | | Very mild NC | | [6] | |  |  |
|  |  | p.V212M | | 99.5 | | 32.4 | | | ND | | ND | | | Normal | | [23] | |  |  |
|  |  | p.M240K | | 95.4 | | 24.7 | | | 97.7 | | 7.7 | | | Normal | | [14] | |  |  |
|  |  | p.A266S | | 90 | | 9 | | | 104 | | 15 | | | Normal | | [13] | |  |  |
|  |  | p.A266V | | 92 | | 1.4 | | | 100 | | 4.3 | | | Normal | | [18] | |  |  |
|  |  | p.P268L | | 97 | | 1 | | | 87 | | 7 | | | Normal | | [6] | |  |  |
|  |  | p.S269T | | 103 | | 15 | | | ND | | ND | | | Normal | | [39] | |  |  |
|  | | Average | | 100.17 | |  | | | 94.08 | |  | | |  | |  | |  |  |
|  | | SD | | 10.92 | |  | | | 8.25 | |  | | |  | |  | |  |  |

Continuation (Table S1)

**Table S2.** Result of 17 predictors for 51 classical single nucleotide variants (SNVs) on the CYP21A2 gene. The classical group has an enzyme activity of < 10% of the wild-type activity. The genomic SNV nomenclature is based on the human chromatin remodeling 38 (Chr38). Del: deleterious; N: Neutral; Pby: Probably; Psb: Possible; B: Benign; Dse: Disease; Efc: Effect; P-Del: Proxy-deleterious; P-N: Proxy-neutral; Dmg: Damaging; T: Tolerated; Ptg: Pathogenic; Csv: Conserved; V: Variable; NR: no result.

| **Chr38** | **SNP** | **PredictSNP** | **PredictSNP2** | **S3Ds&GO** | **Meta-SNP** | **CADD** | **ConSurf** | **DANN** | **FATHMM** | **MAPP** | **MutPred2** | **PANTHER** | **PhD-SNPg** | **PolyPhen2** | **PROVEAN** | **SIFT** | **SNAP2** | **SNPs&GO** |
| --- | --- | --- | --- | --- | --- | --- | --- | --- | --- | --- | --- | --- | --- | --- | --- | --- | --- | --- |
| g.32038514C>A | p.P31Q | Del | N | Dse | Dse | P-Del | Csv | Del | Dmg | Del | Del | Pby | Ptg | Pby | Del | Del | Efc | N; |
| g.32038591G>A | p.G57R | Del | Del | Dse | Dse | P-Del | Csv | Del | Dmg | Del | Del | Pby | Ptg | Pby | Del | Del | Efc | Dse |
| g.32038616G>A | p.G65E | Del | Del | Dse | Dse | P-Del | Csv | Del | Dmg | Del | Del | Pby | Ptg | Pby | Del | Del | Efc | Dse |
| g.32038752T>C | p.I78T | Del | Del | Dse | N | P-Del | Csv | Del | Dmg | Del | Del | Pby | Ptg | Pby | N | N | N | N |
| g.32038791G>T | p.G91V | Del | Del | Dse | Dse | P-Del | Csv | Del | Dmg | Del | Del | Pby | Ptg | Pby | Del | Del | Efc | Dse |
| g.32039124T>G | p.L108R | Del | N | Dse | Dse | P-Del | Csv | Del | Dmg | Del | Del | Pby | Ptg | Pby | Del | Del | Efc | Dse |
| g.32039142C>T | p.S114F | Del | Del | Dse | Dse | P-Del | Csv | Del | Dmg | Del | Del | Pby | B | Pby | Del | Del | Efc | Dse |
| g.32039169T>C | p.L123P | Del | N | Dse | Dse | P-Del | Csv | Del | T | Del | Del | Pby | Ptg | Pby | Del | Del | Efc | Dse |
| g.32039220T>A | p.V140E | Del | N | Dse | Dse | P-Del | Csv | Del | T | Del | Del | Pby | Ptg | Pby | Del | Del | Efc | Dse |
| g.32039229T>C | p.L143P | Del | N | Dse | Dse | P-Del | Csv | Del | T | Del | Del | Pby | Ptg | Pby | N | N | N | Dse |
| g.32039243T>C | p.C148R | N | N | N | N | P-Del | Csv | N | Dmg | Del | Del | Pby | Ptg | Psb | Del | N | N | N |
| g.32039408T>C | p.L167P | Del | N | Dse | Dse | P-Del | V | Del | T | Del | Del | Pby | Ptg | Pby | Del | Del | Efc | Dse |
| g.32039411T>C | p.L168P | Del | Del | Dse | Dse | P-Del | Csv | Del | Dmg | Del | Del | Pby | B | Pby | N | N | N | Dse |
| g.32039416T>C | p.C170R | Del | N | Dse | Dse | P-Del | Csv | Del | Dmg | Del | Del | Pby | B | Pby | Del | Del | Efc | Dse |
| g.32039423T>A | p.I172N | Del | N | Dse | Dse | P-Del | Csv | Del | Dmg | Del | Del | Pby | Ptg | Pby | Del | Del | Efc | Dse |
| g.32039426T>A | p.I173N | Del | Del | Dse | Dse | P-Del | Csv | Del | Dmg | Del | Del | Pby | Ptg | Pby | Del | Del | Efc | Dse |
| **Chr38**  Continuation (Table S2) | **SNP** | **PredictSNP** | **PredictSNP2** | **S3Ds&GO** | **Meta-SNP** | **CADD** | **ConSurf** | **DANN** | **FATHMM** | **MAPP** | **MutPred2** | **PANTHER** | **PhD-SNPg** | **PolyPhen2** | **PROVEAN** | **SIFT** | **SNAP2** | **SNPs&GO** |
| g.32039443G>A | p.G179R | Del | Del | Dse | Dse | P-Del | Csv | Del | Dmg | Del | Del | Pby | Ptg | Pby | Del | Del | Efc | Dse |
| g.32039797A>G | p.R234G | N | N | Dse | Dse | P-Del | Csv | Del | T | N | Del | Pby | B | Pby | Del | Del | Efc | Dse |
| g.32039807T>A | p.I237N | Del | N | N | Dse | P-Del | V | Del | T | Del | Del | Pby | Ptg | Psb | Del | Del | Efc | Dse |
| g.32039810T>A | p.V238E | Del | N | Dse | Dse | P-Del | Csv | Del | T | Del | Del | Pby | Ptg | Psb | Del | Del | Efc | Dse |
| g.32040111T>G | p.V282G | Del | Del | Dse | Dse | P-Del | Csv | Del | Dmg | Del | Del | Pby | Ptg | Pby | Del | Del | Efc | Dse |
| g.32040113C>A | p.H283N | N | N | Dse | Dse | P-Del | Csv | Del | Dmg | N | N | Pby | Ptg | Pby | Del | Del | N | Dse |
| g.32040140G>A | p.G292S | Del | Del | Dse | Dse | P-Del | Csv | Del | Dmg | Del | Del | Pby | Ptg | Pby | Del | Del | Efc | Dse |
| g.32040140G>C | p.G292R | Del | Del | Dse | Dse | P-Del | Csv | Del | Dmg | Del | Del | Pby | Ptg | Pby | Del | Del | Efc | Dse |
| g.32040140G>T | p.G292C | Del | Del | Dse | Dse | P-Del | Csv | Del | Dmg | Del | Del | Pby | Ptg | Pby | Del | Del | Efc | Dse |
| g.32040144G>A | p.G293D | Del | Del | Dse | Dse | P-Del | Csv | Del | Dmg | Del | Del | Pby | Ptg | Pby | Del | Del | Efc | Dse |
| g.32040167C>T | p.L301F | Del | Del | Dse | Dse | P-Del | Csv | Del | Dmg | N | Del | Pby | B | Pby | Del | Del | Efc | Dse |
| g.32040173T>C | p.W303R | Del | Del | Dse | Dse | P-Del | Csv | Del | Dmg | Del | Del | Pby | Ptg | Pby | Del | Del | Efc | Dse |
| g.32040174G>C | p.W303S | Del | Del | Dse | Dse | P-Del | Csv | Del | Dmg | Del | Del | Pby | Ptg | Pby | Del | Del | Efc | Dse |
| g.32040191C>T | p.L309F | N | Del | N | N | P-Del | Csv | Del | Dmg | N | N | Pby | B | Pby | Del | Del | N | N |
| g.32040427G>A | p.E321K | Del | Del | Dse | Dse | P-Del | Csv | Del | Dmg | Del | Del | Pby | Ptg | Pby | Del | Del | Efc | Dse |
| g.32040490C>T | p.R342W | Del | N | Dse | Dse | P-Del | Csv | Del | Dmg | N | Del | Pby | Ptg | Pby | Del | Del | Efc | Dse |
| g.32040491G>C | p.R342P | Del | N | Dse | Dse | P-Del | Csv | Del | T | Del | Del | Pby | B | Pby | Del | Del | Efc | Dse |
| g.32040520G>A | p.E352K | Del | Del | Dse | Dse | P-Del | Csv | Del | Dmg | Del | Del | Pby | Ptg | Pby | Del | Del | Efc | Dse |
| g.32040530G>A | p.R355H | Del | Del | Dse | Dse | P-Del | Csv | Del | Dmg | Del | Del | Pby | Ptg | Psb | Del | Del | Efc | Dse |
| **Chr38** | **SNP** | **PredictSNP** | **PredictSNP2** | **S3Ds&GO** | **Meta-SNP** | **CADD** | **ConSurf** | **DANN** | **FATHMM** | **MAPP** | **MutPred2** | **PANTHER** | **PhD-SNPg** | **PolyPhen2** | **PROVEAN** | **SIFT** | **SNAP2** | **SNPs&GO** |
| g.32040535C>T | p.R357W | Del | N | Dse | Dse | P-Del | Csv | Del | T | N | Del | Pby | Ptg | Pby | Del | Del | Efc | Dse |
| g.32040536G>A | p.R357Q | Del | Del | Dse | Dse | P-Del | Csv | Del | Dmg | N | N | Pby | Ptg | Pby | Del | Del | Efc | Dse |
| g.32040536G>C | p.R357P | Del | Del | Dse | Dse | P-Del | Csv | Del | Dmg | Del | Del | Pby | Ptg | Pby | Del | Del | Efc | Dse |
| g.32040554C>T | p.A363V | N | N | Dse | N | P-Del | Csv | Del | T | N | N | Pby | Ptg | Pby | N | N | N | N |
| g.32040675G>A | p.G376S | Del | Del | Dse | Dse | P-Del | Csv | Del | Dmg | N | Del | Pby | Ptg | Pby | Del | Del | Efc | Dse |
| g.32040715T>G | p.L389R | Del | Del | Dse | Dse | P-Del | Csv | Del | Dmg | Del | Del | Pby | Ptg | Pby | Del | Del | Efc | Dse |
| g.32040728C>G | p.H393Q | N | N | Dse | N | P-Del | Csv | Del | T | N | Del | Pby | B | B | Del | Del | Efc | N |
| g.32040871C>T | p.R409C | Del | Del | Dse | Dse | P-Del | Csv | Del | Dmg | Del | Del | Pby | Ptg | Psb | Del | Del | Efc | Dse |
| g.32040919G>A | p.G425S | Del | Del | Dse | Dse | P-Del | Csv | Del | Dmg | Del | Del | Pby | Ptg | Pby | Del | Del | Efc | Dse |
| g.32040925C>T | p.R427C | Del | Del | Dse | Dse | P-Del | Csv | Del | Dmg | Del | Del | Pby | Ptg | Pby | Del | Del | Efc | Dse |
| g.32040926G>A | p.R427H | Del | Del | Dse | Dse | P-Del | Csv | Del | Dmg | Del | Del | Pby | Ptg | Pby | Del | Del | Efc | Dse |
| g.32040986T>C | p.L447P | Del | Del | Dse | Dse | P-Del | Csv | Del | Dmg | Del | Del | Pby | B | Pby | Del | Del | Efc | Dse |
| g.32040997A>C | p.T451P | Del | N | N | Dse | P-Del | Csv | Del | T | Del | Del | Pby | Ptg | Psb | Del | Del | N | N |
| g.32041037C>T | p.P464L | N | Del | N | N | P-Del | Csv | Del | Dmg | Del | N | Pby | Ptg | Pby | Del | Del | N | N |
| g.32041097G>A | p.R484Q | Del | Del | N | Dse | P-Del | Csv | Del | Dmg | N | Del | Pby | Ptg | Pby | Del | Del | Efc | N |
| g.32041097G>C | p.R484P | Del | N | N | Dse | P-Del | Csv | Del | Dmg | Del | Del | Pby | Ptg | Pby | Del | Del | Efc | Dse |

Continuation (Table S2)

**Table S3.** Result of 17 predictors for 39 non-classical single nucleotide variants (SNVs) on the CYP21A2. The non-classical group has an enzyme activity between >10% and < 78% of the wild-type activity. The genomic SNV nomenclature is based on the human chromatin remodeling 38 (Chr38). Del: deleterious; N: Neutral; Pby: Probably; Psb: Possible; B: Benign; Dse: Disease; Efc: Effect; P-Del: Proxy-deleterious; P-N: Proxy-neutral; Dmg: Damaging; T: Tolerated; Ptg: Pathogenic; Csv: Conserved; V: Variable; NR: no result.

| **Chr38** | **SNP** | **PredictSNP** | **PredictSNP2** | **S3Ds&GO** | **Meta-SNP** | **CADD** | **ConSurf** | **DANN** | **FATHMM** | **MAPP** | **MutPred2** | **PANTHER** | **PhD-SNPg** | **PolyPhen2** | **PROVEAN** | **SIFT** | **SNP2** | **SNPs&GO** |
| --- | --- | --- | --- | --- | --- | --- | --- | --- | --- | --- | --- | --- | --- | --- | --- | --- | --- | --- |
| g.32038514C>T | p.P31L | N | N | N | N | P-Del | Csv | N | Dmg | Del | Del | Pby | Ptg | B | N | N | N | N |
| g.32038610A>T | p.H63L | N | N | N | Dse | P-Del | V | N | T | Del | Del | Pby | B | B | N | N | Efc | N |
| g.32039118C>T | p.P106L | N | N | N | N | P-N | V | N | T | N | N | Pby | B | B | N | N | Efc | N |
| g.32039160A>G | p.H120R | N | N | N | N | P-Del | Csv | Del | Dmg | Del | N | Pby | Ptg | Pby | Del | Del | Efc | N |
| g.32039165A>C | p.K122Q | Del | N | N | Dse | P-Del | Csv | Del | Dmg | Del | N | Pby | Ptg | Pby | Del | Del | Efc | N |
| g.32039198C>T | p.R133C | Del | N | Dse | Dse | P-Del | V | Del | T | N | N | Pby | Ptg | Pby | Del | Del | Efc | Dse |
| g.32039222G>A | p.E141K | N | N | N | N | P-Del | V | Del | T | N | Del | Pby | Ptg | Psb | N | N | N | N |
| g.32039356C>T | p.R150C | N | Del | Dse | N | P-Del | Csv | Del | Dmg | N | N | Pby | B | Pby | N | N | N | N |
| g.32039357G>C | p.R150P | Del | N | Dse | Dse | P-Del | Csv | Del | Dmg | Del | Del | Pby | Ptg | Pby | N | N | N | Dse |
| g.32039360T>G | p.M151R | Del | N | Dse | Dse | P-Del | Csv | Del | Dmg | Del | Del | Pby | Ptg | Psb | Del | Del | Efc | Dse |
| g.32039444G>C | p.G179A | Del | Del | Dse | Dse | P-Del | Csv | Del | Dmg | Del | N | Pby | Ptg | Pby | Del | Del | Efc | Dse |
| g.32039570T>C | p.Y192H | N | N | N | N | P-N | Csv | N | T | N | N | Pby | B | B | N | N | N | N |
| g.32039580T>A | p.I195N | N | N | Dse | N | P-Del | Csv | Del | T | N | Del | Pby | Ptg | Pby | N | Del | Efc | Dse |
| g.32039770C>T | p.R225W | N | N | N | Dse | P-N | V | N | T | N | Del | Pby | B | B | Del | N | N | N |
| g.32039789T>C | p.I231T | N | N | N | N | P-Del | V | Del | T | N | N | Pby | B | B | N | Del | N | N |
| g.32039798G>A | p.R234K | N | N | Dse | N | P-Del | Csv | Del | Dmg | N | N | Pby | Ptg | Pby | N | Del | Efc | N |
| **Chr38**  Continuation (Table S3) | **SNP** | **PredictSNP** | **PredictSNP2** | **S3Ds&GO** | **Meta-SNP** | **CADD** | **ConSurf** | **DANN** | **FATHMM** | **MAPP** | **MutPred2** | **PANTHER** | **PhD-SNPg** | **PolyPhen2** | **PROVEAN** | **SIFT** | **SNP2** | **SNPs&GO** |
| g.32040110G>T | p.V282L | N | N | N | N | P-Del | Csv | Del | Dmg | N | N | Pby | Ptg | Psb | N | N | N | N |
| g.32040116A>G | p.M284V | N | Del | Dse | N | P-Del | Csv | Del | Dmg | N | N | Pby | Ptg | Pby | Del | Del | Efc | Dse |
| g.32040179G>A | p.V305M | N | Del | N | Dse | P-Del | Csv | Del | Dmg | N | N | Pby | B | Pby | N | Del | N | N |
| g.32040185T>G | p.F307V | N | N | Dse | N | P-Del | Csv | Del | Dmg | N | Del | Pby | B | Pby | Del | Del | N | Dse |
| g.32040434A>G | p.D323G | Del | N | N | Dse | P-Del | V | Del | T | Del | Del | Pby | B | Pby | Del | Del | N | N |
| g.32040485G>A | p.R340H | Del | Del | N | Dse | P-Del | Csv | Del | Dmg | N | Del | Pby | B | Pby | Del | Del | Efc | Dse |
| g.32040541G>A | p.V359I | N | N | N | N | P-Del | Csv | Del | Dmg | N | N | Pby | B | Psb | N | Del | N | N |
| g.32040562C>A | p.H366N | Del | N | Dse | Dse | P-Del | Csv | Del | Dmg | Del | Del | Pby | Ptg | Pby | Del | Del | Efc | N |
| g.32040565C>T | p.R367C | N | N | N | N | P-Del | Csv | Del | T | N | Del | Pby | B | B | N | N | N | N |
| g.32040574C>T | p.R370W | Del | N | Dse | Dse | P-Del | Csv | Del | T | Del | N | Pby | B | Pby | Del | Del | Efc | Dse |
| g.32040575G>A | p.R370Q | N | N | N | N | P-Del | Csv | Del | T | N | N | Pby | B | Psb | N | N | N | N |
| g.32040681G>T | p.D378Y | N | N | N | N | P-Del | Csv | Del | T | N | Del | Pby | B | Psb | Del | N | N | N |
| g.32040692G>C | p.E381D | N | Del | N | N | P-Del | Csv | Del | Dmg | N | N | Pby | B | B | N | Del | Efc | N |
| g.32040723G>A | p.A392T | N | Del | Dse | N | P-Del | Csv | Del | Dmg | Del | N | Pby | B | Pby | N | N | N | Dse |
| g.32040771G>A | p.D408N | N | Del | N | N | P-Del | Csv | Del | Dmg | N | N | Pby | Ptg | Pby | N | Del | N | N |
| g.32040940G>A | p.E432K | Del | Del | Dse | Dse | P-Del | Csv | Del | Dmg | N | Del | Pby | Ptg | Pby | Del | Del | Efc | Dse |
| g.32040950C>T | p.A435V | Del | Del | Dse | Dse | P-Del | Csv | Del | Dmg | Del | Del | Pby | Ptg | Pby | Del | Del | Efc | Dse |
| g.32040998C>T | p.T451M | Del | N | N | N | P-Del | Csv | Del | T | Del | N | Pby | Ptg | Psb | Del | Del | N | N |
| g.32041006C>T | p.P454S | Del | Del | N | N | P-Del | Csv | Del | Dmg | N | N | Pby | Ptg | Pby | Del | Del | N | N |
| **Chr38** | **SNP** | **PredictSNP** | **PredictSNP2** | **S3Ds&GO** | **Meta-SNP** | **CADD** | **ConSurf** | **DANN** | **FATHMM** | **MAPP** | **MutPred2** | **PANTHER** | **PhD-SNPg** | **PolyPhen2** | **PROVEAN** | **SIFT** | **SNP2** | **SNPs&GO** |
| g.32041031T>C | p.L462P | Del | Del | N | Dse | P-Del | Csv | Del | Dmg | Del | Del | Pby | Ptg | Pby | Del | Del | N | N |
| g.32041068G>T | p.M474I | N | N | N | N | P-N | V | N | T | N | N | Pby | B | B | Del | N | N | N |
| g.32041085G>T | p.R480L | N | N | N | N | P-Del | V | Del | T | N | N | Pby | B | B | N | N | N | N |
| g.32041093C>T | p.P483S | N | Del | N | N | P-Del | Csv | Del | Dmg | N | N | Pby | Ptg | Pby | N | Del | Efc | N |

Continuation (Table S3)

**Table S4**. Result of 17 predictors for 13 neutral single nucleotide variants (SNVs) on the CYP21A2 gene. The neutral group has the enzyme activity known as > 78% of the wild-type activity. The genomic SNV nomenclature is based on the human chromatin remodeling 38 (Chr38). Del: deleterious; N: Neutral; Pby: Probably; Psb: Possible; Dse: Disease; Efc: Effect; P-Del: Proxy-deleterious; P-N: Proxy-neutral; Dmg: Damaging; T: Tolerated; Ptg: Pathogenic; B: Benign; Csv: Conserved; V: Variable; NR: no result.

| **Chr38** | **SNP** | **PredictSNP** | **PredictSNP2** | **S3Ds&GO** | **Meta-SNP** | **CADD** | **ConSurf** | **DANN** | **FATHMM** | **MAPP** | **MutPred2** | **PANTHER** | **PhD-SNPg** | **PolyPhen2** | **PROVEAN** | **SIFT** | **SNP2** | **SNPs&GO** |
| --- | --- | --- | --- | --- | --- | --- | --- | --- | --- | --- | --- | --- | --- | --- | --- | --- | --- | --- |
| g.32038459C>A | p.L13M | N | N | NR | N | P-Del | NR | Del | T | Del | N | NR | B | Pby | N | Del | N | N |
| g.32038468G>A | p.A16T | N | N | NR | N | P-N | NR | N | T | N | N | NR | B | B | N | N | N | N |
| g.32038471C>T | p.R17C | N | N | NR | N | P-N | NR | Del | T | N | N | NR | B | B | N | Del | N | N |
| g.32039109G>A | p.R103K | N | N | N | N | P-N | V | N | T | N | N | NR | B | B | N | N | N | N |
| g.32039386G>A | p.A160T | N | N | N | N | P-N | Csv | N | T | N | N | Pby | B | B | N | N | N | N |
| g.32039548C>G | p.D184E | N | N | N | N | P-N | V | N | T | N | N | Pby | B | B | N | N | N | N |
| g.32039603A>G | p.S203G | N | N | N | N | P-N | V | N | T | N | N | Pby | B | B | N | N | N | N |
| g.32039630G>A | p.V212M | N | N | N | N | P-N | V | Del | T | N | N | Pby | B | B | N | N | N | N |
| g.32039816T>A | p.M240K | N | N | N | N | P-Del | V | N | T | N | N | Pby | Ptg | B | N | N | N | N |
| g.32040062G>T | p.A266S | N | N | N | N | P-N | V | N | T | N | N | Pby | B | B | N | N | N | N |
| g.32040063C>T | p.A266V | N | N | N | N | P-N | V | Del | T | Del | N | Pby | B | B | N | N | N | N |
| g.32040069C>T | p.P268L | N | N | NR | N | P-N | V | N | T | N | N | Pby | B | B | N | N | N | N |
| g.32040072G>C | p.S269T | N | N | NR | N | P-N | V | N | T | NR | N | Pby | B | B | N | N | N | N |

**Table S5**. Single predictors selected for performance analysis with *CYP21A2* variants.

| Single Predictors | Description | Website | Ref. |
| --- | --- | --- | --- |
| CADD | Integrative annotation built based on diverse genomic feature derived from surrounding sequence context, gene model **annotation**, **evolutionary** constraint, **epigenetic** measurements, and **functional** predictions. | https://cadd.gs.washington.edu/ | [40] |
| ConSurf | Algorithm uses phylogenetic relationships among homologous sequences and the specific dynamics of the analyzed sequence with **evolutionary** models to estimate the evolutionary rates of the amino acid of the macromolecules and to map them onto the structure and/or sequence. | https://consurf.tau.ac.il/ | [41] |
| DANN | Deep neural network which takes non-linear relationships among features based on diverse genomic derived from surrounding sequence context, gene model **annotation**, **evolutionary** constraint, **epigenetic** measurements, and **functional** predictions. | https://cbcl.ics.uci.edu/public_data/DANN/ | [42] |
| FATHMM | **Evolutionary** conservation algorithm which uses homologous sequences with species-specific weighting to predict the protein’s tolerance to missense variants. | http://fathmm.biocompute.org.uk/ | [43] |
| MAPP | A statistical framework predictor which uses protein **physicochemical** characteristics of each amino acid position on the **evolutionary** variation. | http://mendel.stanford.edu/sidowlab/downloads/MAPP/index.html | [44] |
| MutPred2 | Machine learning-based to predict amino acid substitution through **evolutionary**, **structural,** and **functional** proprieties. | http://mutpred.mutdb.org/ | [45] |
| PANTHER-PSEP | Predict using **evolutionary** preservation data, measuring though the length of time estimation that a site has been preserved. | http://www.pantherdb.org/tools/csnpScoreForm.jsp | [46] |
| PhD-SNP^g^ | Machine learning algorithm for predicting SNVs in both non-coding and coding regions through **evolutionary data**. | https://snps.biofold.org/phd-snpg/ | [47] |
| PolyPhen-2 | It uses human protein **evolutionary** and **structural** data to predict amino acid substitution effect on the protein stability and functionality. | http://genetics.bwh.harvard.edu/pph2/ | [48] |
| PROVEN | Predict the functional effect through amino acid exchange **evolutionary** data and quality of the neighborhood sequence alignment rather than the target position. | http://provean.jcvi.org/genome_submit_2.php?species=human | [49] |
| SIFT | Predicts through sequence homology algorithm assuming **evolutionary** conserved regions tend to be less tolerant. | https://sift.bii.a-star.edu.sg/www/SIFT4G_vcf_submit.html | [50] |
| SNAP2 | A neural network method based on machine learning to predict the variant effect in the molecular function through **evolutionary** and **structural** protein data with an amino acid substitution matrix of effect probabilities. | https://rostlab.org/services/snap2web/ | [51] |
| SNPs&GO | Predict using **evolutionary** data, profile and gene ontology (biological process, cellular component and molecular function). When protein function is not available, it run PANTHER and PhD-SNP. | https://snps.biofold.org/snps-and-go/snps-and-go.html | [52] |

**Table S6.** Performance of predictor tools tested described in the literature.

| Predictor | PPV | NPV | Se | Sp | Ac | MCC | AUC-ROC | Dataset | Ref. |
| --- | --- | --- | --- | --- | --- | --- | --- | --- | --- |
| Meta-SNP | 0.79 | 0.8 | 0.8 | 0.79 | 0.79 | 0.59 | 0.86 | SwissVar (2009-2012) | [53] |
| PredictSNP |  |  |  |  | 0.642 | 0.281 | 0.7 | Protein Mutant Database (07Mar26) | [54] |
| PredictSNP2 |  |  |  |  | 0.773 | 0.55 | 0.804 | Mendelian diseases (multiple databases) | [55] |
| SNP&GO3d | 0.84 | 0.86 | 0.87 | 0.83 | 0.85 | 0.7 | 0.92 | Derived from Swiss-Prot (2009) | [56] |
| CADD |  |  | 93.6 | 57.1 | 0.85 |  |  | ClinVar (2015) | [40] |
| DANN |  |  |  |  |  |  | 0.95 | ClinVar (2014) | [42] |
| FATHMM (weighted) | 0.85 | 0.8 | 0.78 | 0.87 | 0.82 | 0.65 |  | SwissVar (2012) | [43] |
| MAPP |  |  |  |  | 0.626-0.767 | |  | Experimental studies | [44] |
| MutPred2 | 96 |  | 42.3 | 95.6 |  |  | 84.9 | ClinVar32 (2015) and UniProt80 (2015) | [45] |
| PANTHER-PSEP |  |  |  |  |  |  | 0.721 | Derived from SwissVar | [46] |
| PhD-SNPg | 0.85 | 0.85 | 0.94 | 0.67 | 0.85 | 0.65 | 0.91 | NewClinvar (2016) | [47] |
| PolyPhen-2* |  |  | 0.85 | 0.6015 |  |  | 0.79 | Mutations on the genes BRCA1, MSH2, MLH1 and TP53 | [57] |
| PROVEAN |  |  | 0.78 | 0.79 |  |  |  | UniProt human protein | [49] |
| SIFT 4G |  |  | 0.8 | 0.735 | 0.7732 | 0.53 |  | UniRef90 (2011) | [50] |
| SNAP2 |  |  |  |  | 0.688 | 0.24 |  | Data set consisting of 9,657 variants from 678 human proteins | [51] |
| SNPs&GO | 0.83 | 0.8 | 0.78 | 0.85 | 0.82 | 0.63 |  | Derived from Swiss-Prot (2008) | [52] |

*Data from an article recommended on the original developer article. PPV, positive predictive value; NPV, negative predictive value; Se, sensibility; Sp, specificity; Ac, accuracy; MCC, Matthews' correlation coefficient test.

**2 Reference**

1. Lajic, S.; Nikoshkov, A.; Holst, M.; Wedell, A. *Effects of Missense Mutations and Deletions on Membrane Anchoring and Enzyme Function of Human Steroid 21-Hydroxylase (P450c21)*; 1999;

2. Soardi, F.C.; Barbaro, M.; Lau, I.F.; Lemos-Marini, S.H. v.; Baptista, M.T.M.; Guerra-Junior, G.; Wedell, A.; Lajic, S.; de Mello, M.P. Inhibition of CYP21A2 Enzyme Activity Caused by Novel Missense Mutations Identified in Brazilian and Scandinavian Patients. *The Journal of Clinical Endocrinology & Metabolism* **2008**, *93*, 2416–2420, doi:10.1210/jc.2007-2594.

3. Ohlsson, G.; Muller, J.; Skakkebaek, N.E.; Schwartz, M. Steroid 21-Hydroxylase Deficiency: Mutational Spectrum in Denmark, Three Novel Mutations, and in Vitro Expression Analysis. *Human Mutation* **1999**, *13*, 482–486, doi:10.1002/(SICI)1098-1004(1999)13:6<482::AID-HUMU8>3.0.CO;2-0.

4. Krone, N.; Riepe, F.G.; Grötzinger, J.; Partsch, C.-J.; Sippell, W.G. Functional Characterization of Two Novel Point Mutations in the *CYP21* Gene Causing Simple Virilizing Forms of Congenital Adrenal Hyperplasia Due to 21-Hydroxylase Deficiency. *The Journal of Clinical Endocrinology & Metabolism* **2005**, *90*, 445–454, doi:10.1210/jc.2004-0813.

5. Nunez, B.S.; Lobato, M.N.; White, P.C.; Meseguer, A. Functional Analysis of Four CYP21 Mutations from Spanish Patients with Congenital Adrenal Hyperplasia. *Biochemical and Biophysical Research Communications* **1999**, *262*, 635–637, doi:10.1006/bbrc.1999.1271.

6. de Paula Michelatto, D.; Karlsson, L.; Lusa, A.L.G.; Silva, C.D.M.; Östberg, L.J.; Persson, B.; Guerra-Júnior, G.; Lemos-Marini, S.H.V. de; Barbaro, M.; de Mello, M.P.; et al. Functional and Structural Consequences of Nine *CYP21A2* Mutations Ranging from Very Mild to Severe Effects. *International Journal of Endocrinology* **2016**, *2016*, 1–10, doi:10.1155/2016/4209670.

7. Massimi, A.; Malaponti, M.; Federici, L.; Vinciguerra, D.; Manca Bitti, M.; Vottero, A.; Ghizzoni, L.; Maccarrone, M.; Cappa, M.; Bernardini, S.; et al. Functional and Structural Analysis of Four Novel Mutations of CYP21A2 Gene in Italian Patients with 21-Hydroxylase Deficiency. *Hormone and Metabolic Research* **2014**, *46*, 515–520, doi:10.1055/s-0034-1371864.

8. Barbaro, M.; Soardi, F.C.; Palandi de Mello, M.; Wedell, A.; Lajic, S. Functional Studies of CYP21A2 Mutants Complement Structural and Clinical Predictions of Disease Severity in CAH. *Clinical Endocrinology* **2012**, *76*, 766–768, doi:10.1111/j.1365-2265.2011.04275.x.

9. Robins, T.; Bellanne-Chantelot, C.; Barbaro, M.; Cabrol, S.; Wedell, A.; Lajic, S. Characterization of Novel Missense Mutations in CYP21 Causing Congenital Adrenal Hyperplasia. *Journal of Molecular Medicine* **2007**, *85*, 247–255, doi:10.1007/s00109-006-0121-x.

10. Tardy, V.; Menassa, R.; Sulmont, V.; Lienhardt-Roussie, A.; Lecointre, C.; Brauner, R.; David, M.; Morel, Y. Phenotype-Genotype Correlations of 13 Rare CYP21A2 Mutations Detected in 46 Patients Affected with 21-Hydroxylase Deficiency and in One Carrier. *Journal of Clinical Endocrinology and Metabolism* **2010**, *95*, 1288–1300, doi:10.1210/jc.2009-1202.

11. Grischuk, Y.; Rubtsov, P.; Riepe, F.G.; Grötzinger, J.; Beljelarskaia, S.; Prassolov, V.; Kalintchenko, N.; Semitcheva, T.; Peterkova, V.; Tiulpakov, A.; et al. Four Novel Missense Mutations in the CYP21A2 Gene Detected in Russian Patients Suffering from the Classical Form of Congenital Adrenal Hyperplasia: Identification, Functional Characterization, and Structural Analysis. *The Journal of Clinical Endocrinology & Metabolism* **2006**, *91*, 4976–4980, doi:10.1210/jc.2006-0777.

12. Barbaro, M.; Baldazzi, L.; Balsamo, A.; Lajic, S.; Robins, T.; Barp, L.; Pirazzoli, P.; Cacciari, E.; Cicognani, A.; Wedell, A. Functional Studies of Two Novel and Two Rare Mutations in the 21-Hydroxylase Gene. *Journal of Molecular Medicine* **2006**, *84*, 521–528, doi:10.1007/s00109-006-0043-7.

13. Barbaro, M.; Soardi, F.C.; Östberg, L.J.; Persson, B.; de Mello, M.P.; Wedell, A.; Lajic, S. In Vitro Functional Studies of Rare CYP21A2 Mutations and Establishment of an Activity Gradient for Nonclassic Mutations Improve Phenotype Predictions in Congenital Adrenal Hyperplasia. *Clinical Endocrinology* **2015**, *82*, 37–44, doi:10.1111/cen.12526.

14. Robins, T.; Barbaro, M.; Lajic, S.; Wedell, A. Not All Amino Acid Substitutions of the Common Cluster E6 Mutation in CYP21 Cause Congenital Adrenal Hyperplasia. *The Journal of Clinical Endocrinology & Metabolism* **2005**, *90*, 2148–2153, doi:10.1210/jc.2004-1937.

15. Lajić, S.; Robins, T.; Krone, N.; Schwarz, H.P.; Wedell, A. CYP21 Mutations in Simple Virilizing Congenital Adrenal Hyperplasia. *Journal of Molecular Medicine* **2001**, *79*, 581–586, doi:10.1007/s001090100261.

16. Concolino, P.; Mello, E.; Patrosso, M.C.; Penco, S.; Zuppi, C.; Capoluongo, E. P.H282N and p.Y191H: 2 Novel CYP21A2 Mutations in Italian Congenital Adrenal Hyperplasia Patients. *Metabolism* **2012**, *61*, 519–524, doi:10.1016/j.metabol.2011.08.008.

17. Nikoshkov, A.; Lajic, S.; Vlamis-Gardikas, A.; Tranebjærg, L.; Holst, M.; Wedell, A.; Luthman, H. Naturally Occurring Mutants of Human Steroid 21-Hydroxylase (P450c21) Pinpoint Residues Important for Enzyme Activity and Stability. *Journal of Biological Chemistry* **1998**, *273*, 6163–6165, doi:10.1074/jbc.273.11.6163.

18. Bleicken, C.; Loidi, L.; Dhir, V.; Parajes, S.; Quinteiro, C.; Dominguez, F.; Grötzinger, J.; Sippell, W.G.; Riepe, F.G.; Arlt, W.; et al. Functional Characterization of Three CYP21A2 Sequence Variants (p.A265V, p.W302S, p.D322G) Employing a Yeast Co-Expression System. *Human Mutation* **2009**, *30*, E443–E450, doi:10.1002/humu.20926.

19. Krone, N.; Riepe, F.G.; Grötzinger, J.; Partsch, C.-J.; Brämswig, J.; Sippell, W.G. The Residue E351 Is Essential for the Activity of Human 21-Hydroxylase: Evidence from a Naturally Occurring Novel Point Mutation Compared with Artificial Mutants Generated by Single Amino Acid Substitutions. *Journal of Molecular Medicine* **2005**, *83*, 561–568, doi:10.1007/s00109-005-0655-3.

20. Lajic, S.; Levo, A.; Nikoshkov, A.; Lundberg, Y.; Partanen, J.; Wedell, A. A Cluster of Missense Mutations at Arg356 of Human Steroid 21-Hydroxylase May Impair Redox Partner Interaction. *Human Genetics* **1997**, *99*, 704–709, doi:10.1007/s004390050436.

21. Chiou, S.H.; Hu, M.C.; CHung, B. A Missense Mutation of Ile172 → Asn or Arg356 → Trp Causes Steroid 21-Hydroxylase Deficiency. *Journal of Biological Chemistry* **1990**, *265*, 3549–3552, doi:10.1016/s0021-9258(19)39804-7.

22. Lajić, S.; Clauin, S.; Robins, T.; Vexiau, P.; Blanché, H.; Bellanne-Chantelot, C.; Wedell, A. Novel Mutations in CYP21 Detected in Individuals with Hyperandrogenism. *The Journal of Clinical Endocrinology & Metabolism* **2002**, *87*, 2824–2829, doi:10.1210/jcem.87.6.8525.

23. Brønstad, I.; Breivik, L.; Methlie, P.; Wolff, A.S.B.; Bratland, E.; Nermoen, I.; Løvås, K.; Husebye, E.S. Functional Studies of Novel CYP21A2 Mutations Detected in Norwegian Patients with Congenital Adrenal Hyperplasia. *Endocrine Connections* **2014**, *3*, 67–74, doi:10.1530/ec-14-0032.

24. Xu, C.; Jia, W.; Cheng, X.; Ying, H.; Chen, J.; Xu, J.; Guan, Q.; Zhou, X.; Zheng, D.; Li, G.; et al. Genotype–Phenotype Correlation Study and Mutational and Hormonal Analysis in a Chinese Cohort with 21‐hydroxylase Deficiency. *Molecular Genetics & Genomic Medicine* **2019**, *7*, doi:10.1002/mgg3.671.

25. Krone, N.; Riepe, F.; Partsch, C.-J.; Vorhoff, W.; Brämswig, J.; Sippell, W. Three Novel Point Mutations of the CYP21 Gene Detected in Classical Forms of Congenital Adrenal Hyperplasia Due to 21-Hydroxylase Deficiency. *Experimental and Clinical Endocrinology & Diabetes* **2006**, *114*, 111–117, doi:10.1055/s-2005-872841.

26. Nikoshkov, A.; Lajic, S.; Holst, M.; Wedell, A.; Luthman, H. Synergistic Effect of Partially Inactivating Mutations in Steroid 21-Hydroxylase Deficiency ^1^. *The Journal of Clinical Endocrinology & Metabolism* **1997**, *82*, 194–199, doi:10.1210/jcem.82.1.3678.

27. Concolino, P.; Vendittelli, F.; Mello, E.; Alinovi, C.C.; Minucci, A.; Carrozza, C.; Santini, S.A.; Zuppi, C.; Capoluongo, E. Two Novel CYP21A2 Missense Mutations in Italian Patients with 21-Hydroxylase Deficiency: Identification and Functional Characterisation. *IUBMB Life* **2009**, *61*, 229–235, doi:10.1002/iub.147.

28. Riepe, F.G.; Hiort, O.; Grötzinger, J.; Sippell, W.G.; Krone, N.; Holterhus, P.-M. Functional and Structural Consequences of a Novel Point Mutation in the *CYP21A2* Gene Causing Congenital Adrenal Hyperplasia: Potential Relevance of Helix C for P450 Oxidoreductase-21-Hydroxylase Interaction. *The Journal of Clinical Endocrinology & Metabolism* **2008**, *93*, 2891–2895, doi:10.1210/jc.2007-2646.

29. Taboas, M.; Gómez Acuña, L.; Scaia, M.F.; Bruque, C.D.; Buzzalino, N.; Stivel, M.; Ceballos, N.R.; Dain, L. Functional Studies of p.R132C, p.R149C, p.M283V, p.E431K, and a Novel c.652-2A&gt;G Mutations of the CYP21A2 Gene. *PLoS ONE* **2014**, *9*, e92181, doi:10.1371/journal.pone.0092181.

30. Chu, X.; Ding, H.; Cui, G.; Xu, Y.; Wang, D.W.; He, Y. Functional Consequences of a Novel Point Mutation in the CYP21A2 Gene Identified in a Chinese Han Patient with Nonclassic 21-Hydroxylase Deficiency. *Clinical Endocrinology* **2014**, *80*, 927–928, doi:10.1111/cen.12309.

31. Concolino, P.; Vendittelli, F.; Mello, E.; Minucci, A.; Carrozza, C.; Rossodivita, A.; Giardina, B.; Zuppi, C.; Capoluongo, E. Functional Analysis of Two Rare CYP21A2 Mutations Detected in Italian Patients with a Mildest Form of Congenital Adrenal Hyperplasia. *Clinical Endocrinology* **2009**, *71*, 470–476, doi:10.1111/j.1365-2265.2008.03517.x.

32. Lajić, S.; Clauin, S.; Robins, T.; Vexiau, P.; Blanché, H.; Bellanne-Chantelot, C.; Wedell, A. Novel Mutations in CYP21 Detected in Individuals with Hyperandrogenism. *The Journal of Clinical Endocrinology & Metabolism* **2002**, *87*, 2824–2829, doi:10.1210/jcem.87.6.8525.

33. Khajuria, R.; Walia, R.; Bhansali, A.; Prasad, R. Functional Characterization and Molecular Modeling of the Mutations in CYP21A2 Gene from Patients with Congenital Adrenal Hyperplasia. *Biochimie* **2018**, *149*, 115–121, doi:10.1016/j.biochi.2018.04.012.

34. Helmberg, A.; Tusie-Luna, M.T.; Tabarelli, M.; Kofler, R.; White, P.C. R339H and P453S: CYP21 Mutations Associated with Nonclassic Steroid 21-Hydroxylase Deficiency That Are Not Apparent Gene Conversions. *Molecular Endocrinology* **1992**, *6*, 1318–1322, doi:10.1210/mend.6.8.1406709.

35. Karlsson, L.; de Paula Michelatto, D.; Lusa, A.L.G.; D’Almeida Mgnani Silva, C.; Östberg, L.J.; Persson, B.; Guerra-Júnior, G.; Valente de Lemos-Marini, S.H.; Baldazzi, L.; Menabó, S.; et al. Novel Non-Classic CYP21A2 Variants, Including Combined Alleles, Identified in Patients with Congenital Adrenal Hyperplasia. *Clinical Biochemistry* **2019**, *73*, 50–56, doi:10.1016/j.clinbiochem.2019.07.009.

36. Hsu, N.-C.; Guzov, V.M.; Hsu, L.-C.; Chung, B. Characterization of the Consequence of a Novel Glu-380 to Asp Mutation by Expression of Functional P450c21 in Escherichia Coli. *Biochimica et Biophysica Acta (BBA) - Protein Structure and Molecular Enzymology* **1999**, *1430*, 95–102, doi:10.1016/S0167-4838(98)00271-4.

37. Robins, T.; Bellanne-Chantelot, C.; Barbaro, M.; Cabrol, S.; Wedell, A.; Lajic, S. Characterization of Novel Missense Mutations in CYP21 Causing Congenital Adrenal Hyperplasia. *Journal of Molecular Medicine* **2007**, *85*, 247–255, doi:10.1007/s00109-006-0121-x.

38. Higashi, Y.; Hiromasa, T.; Tanae, A.; Miki, T.; Nakura, J.; Kondo, T.; Ohura, T.; Ogawa, E.; Nakayama, K.; Fujii-Kuriyama, Y. Effects of Individual Mutations in the P-450(C21) Pseudogene on the P-450(C21) Activity and Their Distribution in the Patient Genomes of Congenital Steroid 21-Hydroxylase Deficiency1. *The Journal of Biochemistry* **1991**, *109*, 638–644, doi:10.1093/oxfordjournals.jbchem.a123433.

39. Wu, D.A.; Chung, B.C. Mutations of P450c21 (Steroid 21-Hydroxylase) at Cys428, Val281, and Ser268 Result in Complete, Partial, or No Loss of Enzymatic Activity, Respectively. *Journal of Clinical Investigation* **1991**, *88*, 519–523, doi:10.1172/JCI115334.

40. van der Velde, K.J.; de Boer, E.N.; van Diemen, C.C.; Sikkema-Raddatz, B.; Abbott, K.M.; Knopperts, A.; Franke, L.; Sijmons, R.H.; de Koning, T.J.; Wijmenga, C.; et al. GAVIN: Gene-Aware Variant INterpretation for Medical Sequencing. *Genome Biology* **2017**, *18*, doi:10.1186/s13059-016-1141-7.

41. Ashkenazy, H.; Abadi, S.; Martz, E.; Chay, O.; Mayrose, I.; Pupko, T.; Ben-Tal, N. ConSurf 2016: An Improved Methodology to Estimate and Visualize Evolutionary Conservation in Macromolecules. *Nucleic Acids Research* **2016**, *44*, W344–W350, doi:10.1093/nar/gkw408.

42. Quang, D.; Chen, Y.; Xie, X. DANN: A Deep Learning Approach for Annotating the Pathogenicity of Genetic Variants. *Bioinformatics* **2015**, *31*, 761–763, doi:10.1093/bioinformatics/btu703.

43. Shihab, H.A.; Gough, J.; Cooper, D.N.; Stenson, P.D.; Barker, G.L.A.; Edwards, K.J.; Day, I.N.M.; Gaunt, T.R. Predicting the Functional, Molecular, and Phenotypic Consequences of Amino Acid Substitutions Using Hidden Markov Models. *Human Mutation* **2013**, *34*, 57–65, doi:10.1002/humu.22225.

44. Stone, E.A.; Sidow, A. Physicochemical Constraint Violation by Missense Substitutions Mediates Impairment of Protein Function and Disease Severity. *Genome Research* **2005**, *15*, 978–986, doi:10.1101/gr.3804205.

45. Pejaver, V.; Urresti, J.; Lugo-Martinez, J.; Pagel, K.A.; Lin, G.N.; Nam, H.J.; Mort, M.; Cooper, D.N.; Sebat, J.; Iakoucheva, L.M.; et al. Inferring the Molecular and Phenotypic Impact of Amino Acid Variants with MutPred2. *Nature Communications* **2020**, *11*, doi:10.1038/s41467-020-19669-x.

46. Tang, H.; Thomas, P.D. PANTHER-PSEP: Predicting Disease-Causing Genetic Variants Using Position-Specific Evolutionary Preservation. *Bioinformatics* **2016**, *32*, 2230–2232, doi:10.1093/bioinformatics/btw222.

47. Capriotti, E.; Fariselli, P. PhD-SNPg: A Webserver and Lightweight Tool for Scoring Single Nucleotide Variants. *Nucleic Acids Research* **2017**, *45*, W247–W252, doi:10.1093/nar/gkx369.

48. Adzhubei, I.; Jordan, D.M.; Sunyaev, S.R. Predicting Functional Effect of Human Missense Mutations Using PolyPhen‐2. *Current Protocols in Human Genetics* **2013**, *76*, doi:10.1002/0471142905.hg0720s76.

49. Choi, Y.; Sims, G.E.; Murphy, S.; Miller, J.R.; Chan, A.P. Predicting the Functional Effect of Amino Acid Substitutions and Indels. *PLoS ONE* **2012**, *7*, doi:10.1371/journal.pone.0046688.

50. Vaser, R.; Adusumalli, S.; Leng, S.N.; Sikic, M.; Ng, P.C. SIFT Missense Predictions for Genomes. *Nature Protocols* **2016**, *11*, 1–9, doi:10.1038/nprot.2015.123.

51. Hecht, M.; Bromberg, Y.; Rost, B. Better Prediction of Functional Effects for Sequence Variants. *BMC Genomics* **2015**, *16*, doi:10.1186/1471-2164-16-S8-S1.

52. Calabrese, R.; Capriotti, E.; Fariselli, P.; Martelli, P.L.; Casadio, R. Functional Annotations Improve the Predictive Score of Human Disease-Related Mutations in Proteins. *Human Mutation* **2009**, *30*, 1237–1244, doi:10.1002/humu.21047.

53. Capriotti, E.; Altman, R.B.; Bromberg, Y. Collective Judgment Predicts Disease-Associated Single Nucleotide Variants. *BMC Genomics* **2013**, *14 Suppl 3*, doi:10.1186/1471-2164-14-s3-s2.

54. Bendl, J.; Stourac, J.; Salanda, O.; Pavelka, A.; Wieben, E.D.; Zendulka, J.; Brezovsky, J.; Damborsky, J. PredictSNP: Robust and Accurate Consensus Classifier for Prediction of Disease-Related Mutations. *PLoS Computational Biology* **2014**, *10*, doi:10.1371/journal.pcbi.1003440.

55. Bendl, J.; Musil, M.; Štourač, J.; Zendulka, J.; Damborský, J.; Brezovský, J. PredictSNP2: A Unified Platform for Accurately Evaluating SNP Effects by Exploiting the Different Characteristics of Variants in Distinct Genomic Regions. *PLoS Computational Biology* **2016**, *12*, doi:10.1371/journal.pcbi.1004962.

56. Capriotti, E.; Altman, R.B. Improving the Prediction of Disease-Related Variants Using Protein Three-Dimensional Structure. *BMC Bioinformatics* **2011**, *12*, doi:10.1186/1471-2105-12-S4-S3.

57. Hicks, S.; Wheeler, D.A.; Plon, S.E.; Kimmel, M. Prediction of Missense Mutation Functionality Depends on Both the Algorithm and Sequence Alignment Employed. *Human Mutation* **2011**, *32*, 661–668, doi:10.1002/humu.21490.
